# G521 is the gatekeeper and a key transmembrane domain contact residue of *Candida albicans* Cdr1

**DOI:** 10.1128/mbio.03746-25

**Published:** 2026-02-26

**Authors:** Mengcun Zhao, Masakazu Niimi, Kyoko Niimi, Ariya Chindamporn, Susumu Kajiwara, Michele Bonus, Holger Gohlke, Lutz Schmitt, Joel D. A. Tyndall, Richard D. Cannon, Erwin Lamping

**Affiliations:** 1 Faculty of Dentistry, Sir John Walsh Research Institute, University of Otago 247194, Dunedin, New Zealand; 2 School of Life Science and Technology, Tokyo Institute of Technology 98406, Yokohama, Japan; 3 Mycology Unit, Department of Microbiology, Faculty of Medicine, Chulalongkorn University 543913, Bangkok, Thailand; 4 Institute for Pharmaceutical and Medicinal Chemistry, Heinrich Heine University Düsseldorf, Düsseldorf, Germany; 5 Institute of Bio- and Geosciences (IBG-4: Bioinformatics), Forschungszentrum Jülich, Jülich, Germany; 6 Institute of Biochemistry, Heinrich Heine University Düsseldorf 229881, Düsseldorf, Germany; 7 School of Pharmacy, University of Otago 170166, Dunedin, New Zealand; University of Wisconsin-Madison, Madison, Wisconsin, USA

**Keywords:** *Candida albicans*, multidrug resistance, Cdr1, ABC transporter, PDR transporter, efflux pump inhibitor, efflux mechanism, entry gate

## Abstract

*Candida albicans* Cdr1 plays an important role in antifungal drug resistance through the extrusion of structurally diverse substrates. However, the molecular basis of its substrate preference remains unclear. A G521R mutation at the center of transmembrane segment 1 (TMS1) was previously found to impair the efflux of large substrates and to confer resistance to large efflux pump inhibitors, suggesting a role for G521 in regulating substrate and inhibitor access. To explore the structural and functional basis of this phenotype, we analyzed a series of *Saccharomyces cerevisiae* strains lacking ABC transporters and overexpressing Cdr1 with G521 replaced by residues of varying size and polarity. Increasing side chain bulk at position 521 progressively reduced transport of larger substrates but had less deleterious, or even positive, effects on the transport of smaller compounds, supporting a size-selective gating function for the G521 residue. Due to the asymmetric nature of Cdr1, no opening exists at the equivalent TMS7 region on the opposite side of the transporter. Further biochemical characterization revealed that substitutions at G521 alter ATP-binding and turnover rates, indicating that this residue also modulates the ATPase activity at the catalytically active nucleotide-binding site and is, therefore, a key regulatory element during the Cdr1 transport cycle. Suppressor screening identified second-site mutations that restored efflux in bulky G521 mutants by reshaping important transmembrane domain contact regions. These findings defined G521 as the gatekeeper residue that couples substrate selection at the entry gate to the large conformational changes experienced by Cdr1 during the transport cycle.

**IMPORTANCE:**

*Candida albicans* is a major fungal pathogen that can cause life-threatening invasive infections in immunocompromised individuals, and the multidrug ABC transporter Cdr1 plays a key role in its antifungal resistance. While previous studies have identified the transporter’s broad substrate specificity, the structural basis underlying substrate selection remains poorly understood. In this study, we identified a key amino acid residue in transmembrane segment 1 with two important biological functions: (i) as a gatekeeper and (ii) as a key transmembrane domain contact residue affecting ATP binding and hydrolysis at the catalytically active composite nucleotide-binding site 2 just underneath the efflux pump entry gate between transmembrane segments 1 and 11. This work provides a critical understanding of how substrates and inhibitors access the Cdr1 binding cavity and how ATP binding and hydrolysis are coupled to substrate transport. These discoveries open new avenues for the development of next-generation antifungal efflux pump inhibitors.

## INTRODUCTION

ATP-binding cassette (ABC) transporters constitute one of the largest protein superfamilies found in all domains of life (1). These integral membrane proteins mediate active transport, export, and import of a wide variety of substrates across cellular membranes using the energy derived from ATP hydrolysis (2). ABC transporters coordinate critical physiological functions, including cellular signaling, xenobiotic detoxification, and nutrient acquisition, in both prokaryotic and eukaryotic cells (3–5). The overexpression of multidrug resistance ABC transporters constitutes a key mechanism driving chemotherapeutic failure in cancer treatment and the antimicrobial resistance of many important bacterial and fungal pathogens (6–9).

Overexpression of *Candida* drug resistance 1 (Cdr1), a pleiotropic drug resistance (PDR) ABC transporter, is a major antifungal resistance mechanism in the opportunistic fungal pathogen *Candida albicans* (10) through the extrusion of many structurally unrelated cytotoxic compounds, including azole antifungals (11, 12). Cdr1 is a type V ABC transporter of the ABCG subfamily whose members are characterized by their reverse topology, with each of the two nucleotide-binding domains (NBDs) preceding a transmembrane domain (TMD), forming a [NBD-TMD]_2_ domain arrangement. Each TMD contains six transmembrane segments (TMSs). Full-size fungal PDR transporters are characterized by their asymmetric nature, with only one of the two composite ATP-binding sites retaining catalytic activity (13, 14).

In the past decade, several high-resolution structures of eukaryotic ABCG transporters have been determined. This includes the human sterol transporter ABCG5/G8 (15) and a variety of structures for the human multidrug transporter ABCG2, capturing the transporter in multiple functional states, including inward-facing conformations bound to substrates or inhibitors (15–17), an ATP-bound post-translocation state (17), an apo-closed resting state (18), and catalytic intermediates under turnover conditions (19). The cryo-EM structures for Cdr1 and its *Saccharomyces cerevisiae* Pdr5 homolog have also recently been determined (20, 21). Yet, a number of important questions remain unanswered: What causes the broad substrate specificity of Cdr1? What distinguishes an efflux pump substrate from an inhibitor? How do substrates and inhibitors access and interact with the binding cavity? And how are substrates extruded into the extracellular space?

*Candida albicans* Cdr1 and Cdr2, as well as *S. cerevisiae* Pdr5 and Snq2, exhibit significant differences in substrate specificity and inhibitor sensitivity, despite their rather high similarities of 92%, 73%, and 60% with Cdr1, respectively (22, 23). While Cdr1 and Pdr5 transport a broad range of substrates, Snq2 preferentially exports smaller compounds (24, 25). Moreover, Cdr1, Cdr2, and Pdr5 confer distinct resistance profiles to pump inhibitors such as FK506 (tacrolimus), FK520 (ascomycin), beauvericin, enniatin B, and milbemycin α25 (26–30). To elucidate the structural basis of these phenotypes, site-directed mutagenesis and resistance-based mutant screening have proved effective in identifying functionally critical efflux pump residues (31).

There is currently no consensus on (i) whether ABCG or PDR transporter substrates and inhibitors access the binding pocket from the cytoplasm or the inner leaflet of the bilayer and (ii) whether substrates are extruded into the extracellular space or simply flipped from the inner to the outer leaflet of the bilayer (32–34). In Cdr1, glycine 521, positioned centrally within TMS1 and facing TMS11, appears to be a critical residue governing substrate and inhibitor entry (29). Exposure of wild-type Cdr1 to resazurin—a known substrate of Snq2 but not Cdr1—induced the emergence of G521S and G521D substitutions, indicating that this residue may contribute to the substrate specificity of Cdr1 and Snq2 (35). The G521R substitution was also recently identified as a mutation able to restore proper folding and efflux pump function to an inactive Cdr1 variant. This variant had all nine N-terminal cysteines—including the two key disulfide bond-forming cysteines in extracellular loop 3 (EL3)—replaced with serine. This finding indicated that G521 may also help stabilize important interactions between the TMD and the extracellular domain (36).

In this study, we characterized the effects of different amino acid substitutions at position 521 on the substrate transport and the ATPase function of Cdr1.

## RESULTS

### Structural consequences of arginine substitution at G521

**Fig 1:**
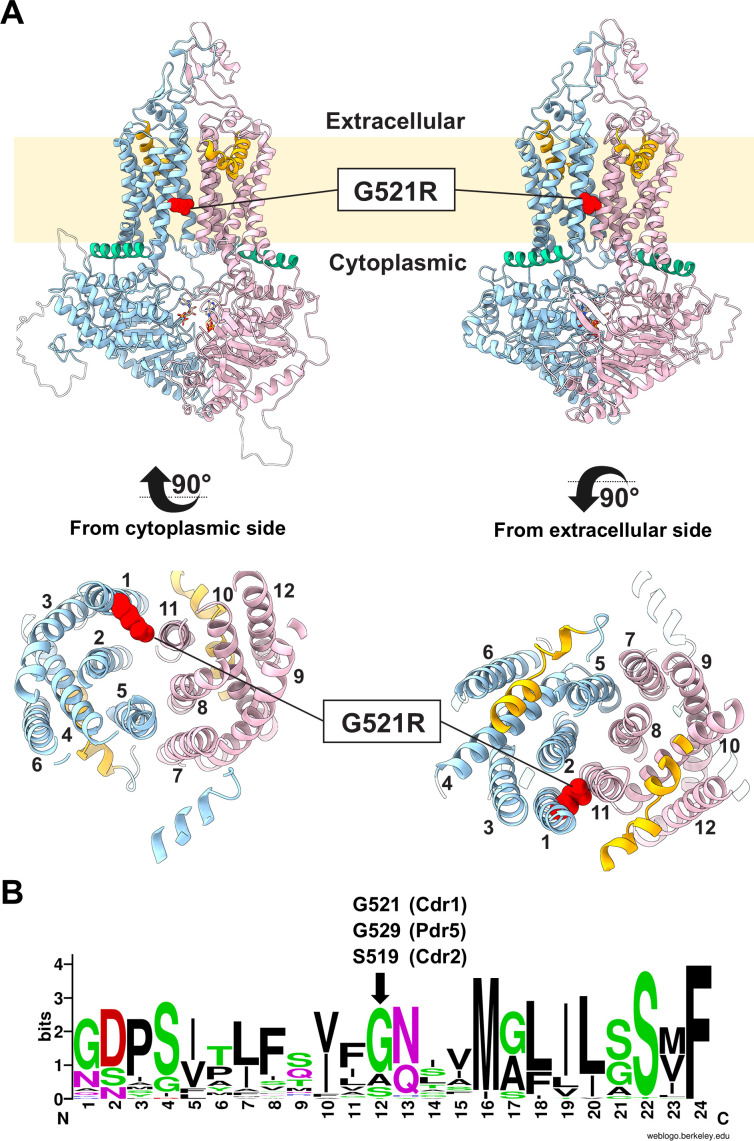
(**A**) Cartoon models of Cdr1 illustrating the conformational transition between the inward-facing (ATP/ADP-bound; left) and the outward-facing (ATP/ADP-vanadate-bound; right) conformation. TMD1 and NBD1 are in cyan, and TMD2 and NBD2 are in pink. Elbow helices (also known as TMS5b/c and TMS11b/c or re-entry helices) and connecting helices are shown in orange and green, respectively. The Cdr1-G521R substitution at the center of TMS1 is shown in red, with the membrane-bilayer boundaries shown in pale yellow. The bottom figures provide orthogonal views of the TMD regions of the inward-facing conformation viewed from the cytoplasm (left) and the outward-facing conformation viewed from the extracellular space (right). (**B**) WebLogo (38) diagram illustrating the sequence conservation of TMS1 residues (i.e., Cdr1-G510 to -F533) among 42 cluster A PDR transporters (13). The black arrow indicates the position of Cdr1-G521, Pdr5-G529, and *C. albicans* Cdr2-S519.

Five Cdr1 models were generated by homology modeling using the *S. cerevisiae* Pdr5 templates (21): inward-open (apo- and ATP/ADP-bound states) and outward-open (ATP/ADP-vanadate-bound state) conformations for Cdr1 and the ATP/ADP and ATP/ADP-vanadate-bound states for the Cdr1-G521R variant. Cdr1 homology models based on Pdr5 structures were used because they provided models for both the inward-open and the outward-open conformations of Cdr1. A comparison of the apo-state model with the recent experimental Cdr1 cryo-EM structure (20) demonstrated good agreement, with a Cα root-mean-square deviation (RMSD) of 1.78 Å and a template-modeling score (37) of 0.98 across 1,333 aligned residues (Fig. S1), which was strong validation of our modeling approach. Cdr1 models predicted that substituting the compact glycine 521 residue with the bulky, charged arginine may introduce a constriction between TMS1 and TMS11 (Fig. 1A). Sequence alignments of 42 cluster A PDR transporters (Fig. 1B) revealed a strong preference for small residues (Gly, Ser, Ala) at the *C. albicans* Cdr1 521 position. Pdr5 also has a glycine (G529) at this position, but *C. albicans* Cdr2 (S519), another cluster A PDR transporter, and *S. cerevisiae* Snq2 (S528), a cluster D PDR transporter (13), have a serine at this position.

### Isolation and generation of *S. cerevisiae* strains overexpressing Cdr1-G521 variants

**Fig 2:**
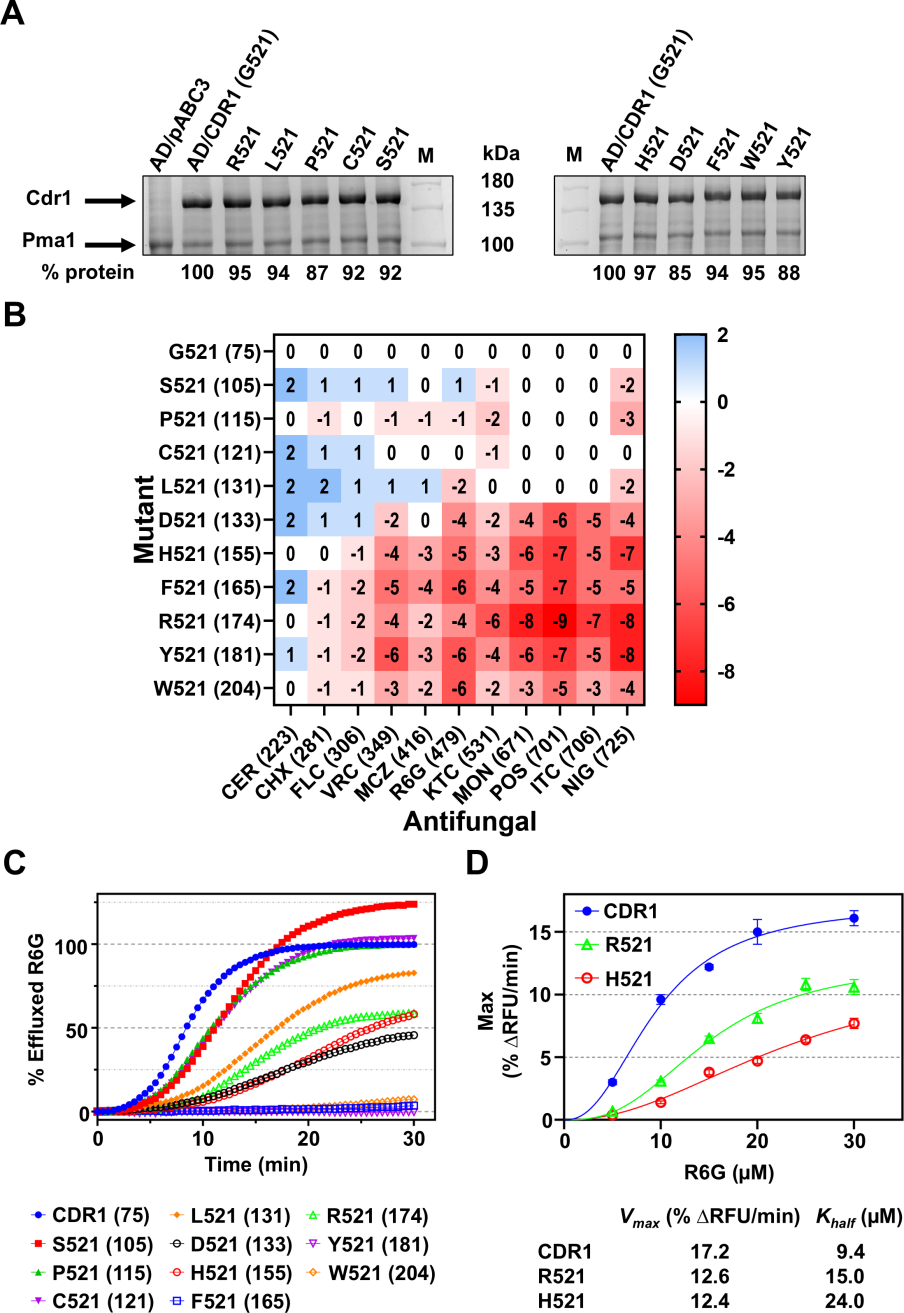
(**A**) SDS-PAGE of 20 μg crude PM proteins from AD cells overexpressing 11 Cdr1-G521 variants, including wild-type Cdr1 and the negative control strain AD/pABC3. Arrows indicate the 170 kDa Cdr1 and the 100 kDa Pma1 bands. M = molecular weight markers. The % Cdr1 expression levels relative to wild-type Cdr1 are listed underneath the images. (**B**) Heatmap of log_2_-transformed fold-increased (blue) or -decreased (red) MIC values of 11 antifungals for AD strains overexpressing wild-type Cdr1 (the reference AD/CDR1B-G521 strain) or 10 different Cdr1-G521 variants. Strains and antifungals are ordered from top to bottom and from left to right according to their molecular weights, which are given in brackets. A color scale depicting the range of fold-increased (0 to 2 = wild-type level to fourfold increased) and fold-decreased (0 to −9 = wild-type level to 512-fold decreased) MIC values is shown to the right of the heatmap. CER: cerulenin, CHX: cycloheximide, FLC: fluconazole, VRC: voriconazole, MCZ: miconazole, R6G: rhodamine 6G, KTC: ketoconazole, MON: monensin, POS: posaconazole, ITC: itraconazole, NIG: nigericin. (**C**) Glucose-dependent R6G efflux by whole cells expressing the 10 Cdr1-G521 variants or wild-type Cdr1. Extracellular R6G fluorescence was measured at 30 s intervals post glucose addition (t = 0). Data are the means of two biological replicates. The R6G efflux values were normalized by (i) subtracting the background signal of the negative control strain AD/pABC3, (ii) adjusting for the slightly different Cdr1 expression levels, and (iii) setting the extracellular R6G levels obtained by Cdr1-G521-expressing cells after 30 min incubation in the presence of glucose to 100%. The molecular weights (Da) of the residues at position 521 are provided in brackets next to individual residues. (**D**) Graph of the maximum R6G efflux rates of whole cells of the positive control strain AD/CDR1B (blue circles) and the R521 (green triangles) and H521 (red circles) variants plotted against the R6G concentrations used to load the cells before initiating R6G efflux from cells with the addition of glucose. The data represent the means ± SD of three biological replicates. Kinetic parameters *V*_max_ and *K*_half_ for each strain are tabulated adjacent to the figure.

To elucidate the role of G521, we isolated four itraconazole (ITC)-resistant “suppressor” mutants of the *S. cerevisiae* AD (AD1-8u^-^) strain overexpressing Cdr1-G521R (AD/CDR1B-G521R), and we also constructed a series of AD strains overexpressing G521 variants with progressively bulkier residues (D, H, F, Y, W). All yeast strains generated in this study are listed in Table 1. The four ITC-resistant G521R suppressor mutants each harbored a mutation within the *CDR1* ORF. Intriguingly, each mutation replaced R521 with an amino acid with a smaller side chain (S, C, L, P), effectively restoring wild-type Cdr1 ITC transport (Fig. 2B; Table S1). SDS-PAGE of crude plasma membrane (PM) preparations of these strains demonstrated similar expression levels to wild-type Cdr1 (Fig. 2A).

**TABLE 1:** Yeast strains used in this study

Strain	Genotype or description	Source
AD (AD1-8u^-^)	*MATa, PDR1–3, ura3, his1,* Δ*yor1::hisG,* Δ*snq2::hisG,* Δ*pdr5::hisG,* Δ*pdr10::hisG,* Δ*pdr11::hisG,* Δ*ycf1::hisG,* Δ*pdr3::hisG,* Δ*pdr15::hisG*	(39)
ADΔ	AD1-8u^-^, Δ*ura3*	(30)
ADΔΔ	AD1-8u^-^, Δ*ura3,* Δ*his1*	(40)
AD/pABC3	AD1-8u^-^, Δ*pdr5::pABC3* (empty vector control)	(30)
ADΔ/pABC3	ADΔ, Δ*pdr5::pABC3* (empty vector control)	(30)
ADΔΔ/pABC3	ADΔΔ, Δ*pdr5::pABC3* (empty vector control)	(36)
AD/CDR1B	AD1-8u^-^, Δ*pdr5::pABC3-CDR1B*	(30)
ADΔΔ/CaCDR1A-GH	ADΔΔ, Δ*pdr5::CaCDR1A-GFP-HIS*	(41)
ADΔΔ/CaCDR1A-E1027Q-GH	ADΔΔ, Δ*pdr5::CaCDR1A-E1027Q-GFP-HIS*	(41)
AD/CDR1B-G521R	Milbemycin α25-resistant CDR1B mutant	(29)
-R521L	ITC-resistant CDR1B-R521 suppressor mutant	This study
-R521P	ITC-resistant CDR1B-R521 suppressor mutant	This study
-R521C	ITC-resistant CDR1B-R521 suppressor mutant	This study
-R521S	ITC-resistant CDR1B-R521 suppressor mutant	This study
ADΔ/CDR1B-G521D	PCR-based site-directed mutagenesis	This study
ADΔ/CDR1B-G521F	PCR-based site-directed mutagenesis	This study
-F521/D511V	ITC-resistant CDR1B-F521 suppressor mutant	This study
-F521/A549V	ITC-resistant CDR1B-F521 suppressor mutant	This study
-F521/T658P	ITC-resistant CDR1B-F521 suppressor mutant	This study
-F521/G672R	R6G-resistant CDR1B-F521 suppressor mutant	This study
ADΔ/CDR1B-G521H	PCR-based site-directed mutagenesis	This study
-H521/A548G	R6G-resistant CDR1B-H521 suppressor mutant	This study
-H521/C712S	R6G-resistant CDR1B-H521 suppressor mutant	This study
-H521/L782F	ITC-resistant CDR1B-H521 suppressor mutant	This study
-H521/L1225I	ITC-resistant CDR1B-H521 suppressor mutant	This study
ADΔ/CDR1B-G521W	PCR-based site-directed mutagenesis	This study
-W521C	ITC-resistant CDR1B-W521 suppressor mutant	This study
-W521S	ITC-resistant CDR1B-W521 suppressor mutant	This study
-W521/R546G	R6G-resistant CDR1B-W521 suppressor mutant	This study
-W521/A549V	ITC-resistant CDR1B-W521 suppressor mutant	This study
-W521/V649F	ITC-resistant CDR1B-W521 suppressor mutant	This study
ADΔ/CDR1B-G521Y	PCR-based site-directed mutagenesis	This study
-Y521/G672A	R6G-resistant CDR1B-Y521 suppressor mutant	This study

### The size of residue 521 as well as the size of the substrate determines the transport efficiency of individual efflux pump substrates

The susceptibilities of the Cdr1-G521 variants to 11 antifungals (a list of compounds and their abbreviations is provided in the Fig. 2B legend), the molecular weights (MWs) of which ranged from 223 to 725 Da, representing a broad spectrum of Cdr1 substrates, were determined. The susceptibility of the variants is a proxy for substrate transport. AD/CDR1B-G521R exhibited progressively impaired transport of larger substrates (Fig. 2B), confirming previous observations (29). Notably, four Cdr1-G521R suppressor mutants (i.e., Cdr1-S/P/C/L/521) restored ITC transport and broadly recovered efflux activity for other large substrates (MWs 400–725 Da) (Fig. 2B). Most of these mutants, apart from Cdr1-P521, were even more efficient (two to four times) than wild-type Cdr1 in transporting the smallest test compounds CER (223 Da), CHX (281 Da), and FLC (306 Da), whereas Cdr1-P521 displayed an identical (CER, FLC) or slightly reduced (twofold; CHX, VRC) transport capacity (Fig. 2B).

Larger substitutions (D/H/F/R/Y/W521 variants) resulted in much more severe transport defects, especially for larger substrates (MW ≥479 Da), the MICs of which were 4–512 times lower than for wild-type Cdr1 (Fig. 2B). However, their small molecule transport capacity remained largely unaffected or was even two- (Y521) or fourfold (F521) improved for the smallest substrate, CER (Fig. 2B). These results confirmed that G521 may indeed be the gatekeeper residue that controls the entry of Cdr1 efflux pump substrates. Apart from the two smallest substrates, CER and CHX, there was a reasonably good correlation between the degree of transport impairment and the increasing MW of residue 521 (Fig. S2). Notably, D521 (133 Da) and L521 (131 Da), despite the similar MWs of the substituted amino acids, differed quite significantly in their efflux pump phenotype (Fig. 2B). Substitutions with S, C, L, F, and W at position 521 showed moderate correlations between reduced transport and increasing substrate MW (*R*² = 0.43–0.67, Fig. S3), which was even more pronounced for charged residues D, H, and R (*R*² = 0.82–0.92, Fig. S3). In contrast, P521 showed no such correlation (*R*² = 0.04).

### Whole-cell R6G transport by the Cdr1-G521 variants also displays a size-dependent transport phenotype

R6G, a fluorescent substrate of Cdr1, enables quantification of efflux pump activity in whole cells (42). The R6G transport of Cdr1-G521 variants was monitored at 30 s intervals over a 30 min time period (Fig. 2C). Three kinetic parameters were used to quantify R6G efflux activity: extracellular R6G fluorescence at equilibrium (EQ), maximum transport rate (MAX; expressed as %ΔRFU/min), and activation half-time (t_1/2_; time required to reach 50% MAX). Parameter comparisons are shown in Table 2. Wild-type Cdr1 exhibited the fastest activation (t_1/2_ = 5.5 min) and the highest maximum transport rate (11 %ΔRFU/min). The Y521 variant showed no detectable efflux activity, while F521 and W521 displayed minimal transport, consistent with low R6G MICs (1 mg/L, Table 2). The smaller P521 and C521 variants achieved comparable R6G equilibria to wild-type Cdr1, in line with comparable MICs, and the S521 variant achieved a ~25% higher EQ, consistent with its twofold higher MIC than wild-type Cdr1 (Fig. 2B and C; Table 2).

**TABLE 2:** Kinetic properties of R6G transport and MIC values of AD/CDR1B and /CDR1B-G521 variants

Strain	MW of residue 521 (Da)	% Cdr1 expression	EQ^*a*^ (%RFU^*a*^)	MAX^*a*^ (%ΔRFU/min)	t_1/2_^*a*^ (min)	MIC of R6G (mg/L)
CDR1	75	100	101 ± 2.5^*b*^	11 ± 0.5	5.5 ± 0.4	64
S521	105	92	125 ± 6.1	11 ± 1.5	8.6 ± 1.5	128
P521	115	87	103 ± 5.8	8.3 ± 1.1	6.7 ± 0.4	32
C521	121	92	106 ± 8.1	8.7 ± 0.3	7.5 ± 1.3	64
L521	131	94	86 ± 2.8	5.6 ± 0.3	10 ± 2.3	16
D521	133	85	51 ± 1.4	2.5 ± 0.3	11 ± 0.9	4
H521	155	97	67 ± 1.4	3.5 ± 0.2	14 ± 1.9	2
F521	165	94	4.5 ± 1.3	0.13 ± 0.01	21 ± 3.7	1
R521	174	95	59 ± 1.3	4.7 ± 0.1	11 ± 0.2	4
Y521	181	88	–^*c*^	–	–	1
W521	204	95	11 ± 5.0	0.6 ± 0.2	20 ± 2.4	1

^*a*^ EQ (equilibrium of extracellular R6G), MAX (maximum slope [rate of R6G efflux]), and t_1/2_ (time to reach half maximum R6G efflux rate) are kinetic parameters of the R6G efflux activity of whole cells overexpressing the indicated Cdr1 variants (RFU, relative fluorescence units).

^*b*^ Values are the means of two independent experiments (± SDs) accounting for the different Cdr1 expression levels.

^*c*^ –, no detectable R6G transport.

Good correlations (*R*^2^ = 0.68–0.93) were observed between the kinetic parameters and both the R6G MICs and the MWs of the G521 variants. The best correlations with R6G MICs and MWs (*R*^2^ = 0.93 or 0.76) were obtained for the maximum transport rates (Fig. S4). The whole-cell R6G maximum transport rate kinetics of Cdr1-G521 and the R521 and H521 variants revealed an allosteric sigmoidal behavior (Fig. 2D). Cdr1 has been shown to transport two substrate molecules simultaneously (27). Perhaps there is a low-affinity R6G binding site that stimulates binding of a second molecule nearby, causing multiple occupancy at >5 µM–10 µM R6G. The R521 and H521 variants showed reduced *V*_max_ and elevated *K*_half_, confirming that bulkier, charged substitutions at G521 impair both transport capacity and substrate affinity. The R6G efflux activities were inhibited above 30 μM R6G (data not shown).

### Starvation, freeze-thawing, and storage times all affect the Cdr1-specific ATPase activity of crude PMs

**Fig 3:**
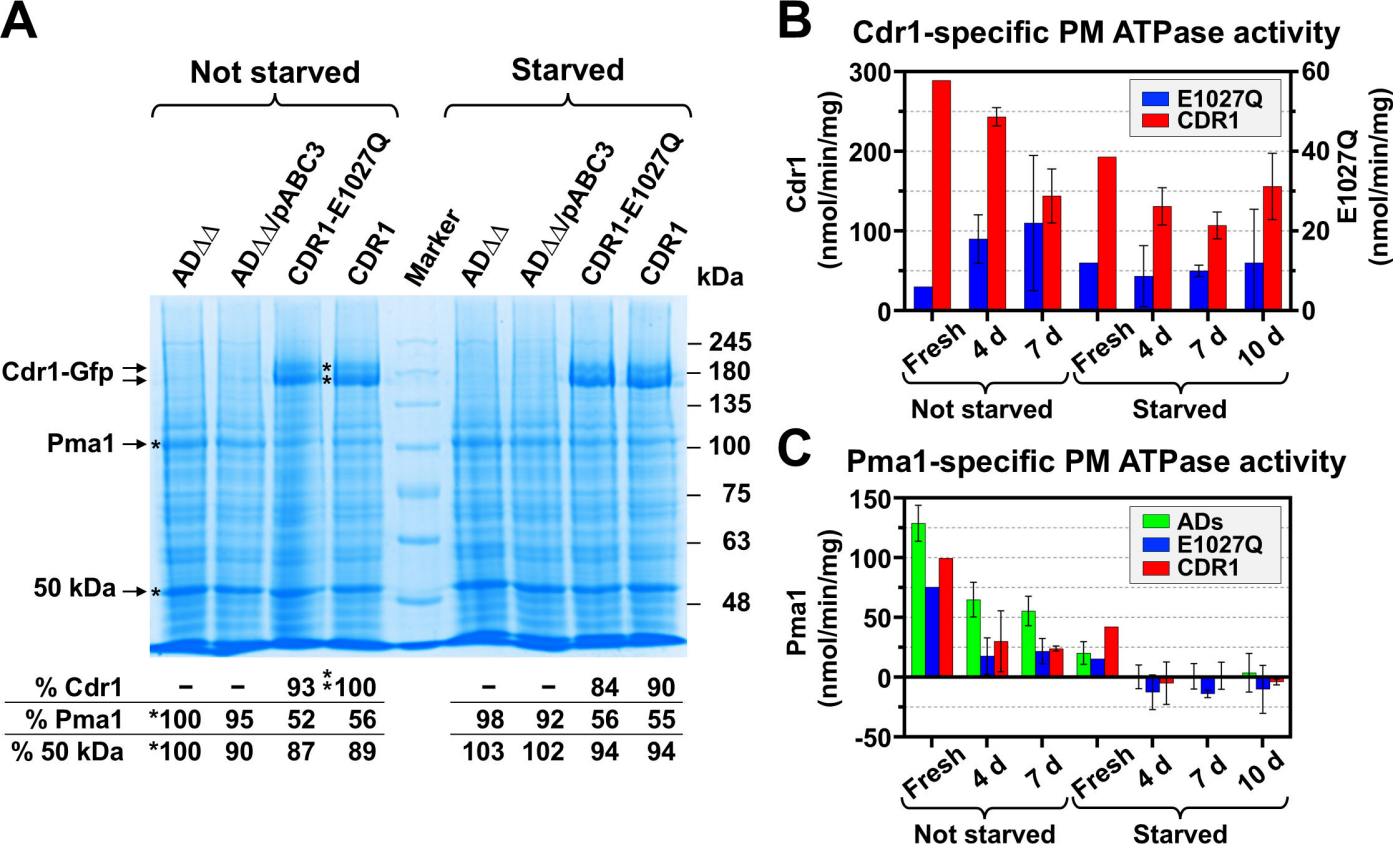
Characterization of crude PMs isolated from logarithmic phase AD1-8u^-^ cells. The following strains were used for these investigations: six hypersusceptible AD1-8u^-^ derivative strains with (AD-pABC3, ADΔ-pABC3, ADΔΔ-pABC3) and without (AD, ADΔ, ADΔΔ) the empty transformation cassette (i.e., with and without the *URA3* marker) and ADΔΔ cells overexpressing either the inactive *CDR1-E1027Q* mutant or wild-type *CDR1* each with a C-terminal GFP-His double tag (44). (**A**) SDS-PAGE of 30 µg crude PM proteins of logarithmic cells of the indicated strains grown in YPD medium, harvested and frozen immediately (left lanes) or starved in ice-cold water for 1 h (lanes to the right of the molecular weight marker) before cell harvest. Arrows indicate the ~200 kDa CaCdr1-GFP-His double band (CaCdr1-GFP-His runs as a double band because most of the C-terminal Gfp tag was not fully denatured even in the presence of 2% SDS and after 5 min denaturation at 50°C), the ~110 kDa Pma1 and the prominent ~50 kDa protein band that was used as a loading control. The % protein expression levels of Cdr1, Pma1, and 50 kDa proteins relative to the bands indicated with * are listed underneath the image. (**B, C**) demonstrate the effects of glucose starvation, the quality (fresh versus frozen), and storage duration (4, 7, and 10 days at −20°C) on (**B**) the Cdr1-specific and (**C**) the Pma1-specific ATPase activities of the six AD1-8u^-^ strains (green bars) and of ADΔΔ cells overexpressing either wild-type *CDR1* (red bars) or *CDR1-E1027Q* (blue bars). Cdr1 = the oligomycin-sensitive (OLI-S) ATPase activity minus the average OLI-S background ATPase activity of the six AD1-8u^-^ control strains; Pma1 = the vanadate-sensitive (VAN-S) ATPase activity minus the OLI-S ATPase activity.

We have developed an efficient small-scale PM isolation protocol for the characterization of Cdr1-specific ATPase activities. As part of the protocol optimization, we discovered that the ATPase activities of PM preparations are sensitive to storage conditions and investigated this further. We used the PM isolation protocol to determine the optimum control strain to provide the background ATPase activity. We measured the PM ATPase activities of six different AD control strains (three with and three without a *URA3* marker, Table 1) and the PM ATPase activities of ADΔΔ cells overexpressing either wild-type Cdr1 or the efflux pump-inactive Cdr1-E1027Q variant. This variant is equivalent to the catalytically inactive Pdr5-E1036Q Walker B2 mutant (43). The results summarized in Fig. 3 and Table 3 demonstrated that (i) overexpression of Cdr1 and Cdr1-E1027Q markedly reduced (~50%) the expression levels (Fig. 3A) and the ATPase activities (Fig. 3C) of the abundant PM proton pump, Pma1; (ii) the Pma1 background ATPase activity was further reduced (by ~80%; Fig. 3C) by glucose starvation and eliminated with just one additional freeze-thaw cycle (Fig. 3C); (iii) overexpression of Cdr1 did not alter the rather low (~5%–10%) oligomycin-sensitive (OLI-S) background ATPase activity of the AD control strains (Table 3); and (iv) The Cdr1-E1027Q variant had residual (~3%–5%) OLI-S ATPase activity that was much more stable than the OLI-S background ATPase activity of the AD strains (Fig. 3B; Table 3). These observations, and the fact that some Cdr1 variants were also resistant to OLI—the reason why we could not use the OLI-S ATPase activities to determine the Cdr1-specific ATPase activities—demonstrated that it does not matter which strain lacking Cdr1 is used as the background to accurately determine the Cdr1-specific ATPase activity of Cdr1 variants. We subtracted the total PM ATPase activity of AD/pABC3 from the total ATPase activities of the Cdr1 variants. These data are presented and discussed in more detail in File S1.

**TABLE 3:** Mean ATPase activities of crude PMs isolated from six AD1-8u^-^ derivative strains (ADs) and two ADΔΔ strains overexpressing either wild-type *CDR1* or the “catalytically inactive” *CDR1-E1027Q* variant^*h*^

Cells	Strain	PMs^*a*^	ATPase activities (nmol/min/mg) (± SD)					
			Total	OLI-S^*b*^	VAN-S^*c*^	VAN-IS	Cdr1^*d*^	Pma1^*e*^
Not starved	ADs^*f*^	Fresh	243 ± 15	43 ± 8	172 ± 13	72 ± 4	–^*i*^	129 ± 15
		4 days	149 ± 16	18 ± 11	83 ± 10	65 ± 10	–	65 ± 14
		7 days	116 ± 8	3 ± 5	58 ± 9	58 ± 6	–	55 ± 12
	E1027Q	Fresh	208 (−)^*g*^	49 (−)	124 (−)	84 (−)	6 (−)	75 (−)
		4 days	136 ± 5	37 ± 8	54 ± 7	82 ± 7	18 ± 6	18 ± 15
		7 days	113 ± 17	25 ± 15	47 ± 4	66 ± 13	22 ± 6	22 ± 11
	CDR1	Fresh	507 (−)	332 (−)	432 (−)	75 (−)	289 (−)	100 (−)
		4 days	365 ± 20	262 ± 20	292 ± 15	73 ± 18	243 ± 11	30 ± 26
		7 days	241 ± 37	146 ± 31	170 ± 29	70 ± 8	144 ± 34	24 ± 2
Starved	ADs	Fresh	106 ± 8	29 ± 2	49 ± 9	57 ± 2	–	20 ± 10
		4 days	79 ± 14	19 ± 8	19 ± 5	59 ± 14	–	0 ± 10
		7 days	78 ± 9	16 ± 8	17 ± 5	61 ± 9	–	1 ± 11
		10 days	66 ± 12	11 ± 11	15 ± 6	50 ± 14	–	4 ± 16
	E1027Q	Fresh	131 (−)	41 (−)	57 (−)	75 (−)	12 (−)	15 (−)
		4 days	92 ± 23	28 ± 15	15 ± 2	76 ± 23	9 ± 7	−13 ± 15
		7 days	87 ± 4	26 ± 2	12 ± 5	75 ± 8	10 ± 2	−14 ± 3
		10 days	78 ± 23	23 ± 17	13 ± 3	65 ± 25	12 ± 13	−10 ± 20
	CDR1	Fresh	334 (−)	221 (−)	264 (−)	70 (−)	193 (−)	42 (−)
		4 days	230 ± 39	150 ± 30	145 ± 34	85 ± 27	131 ± 24	−5 ± 18
		7 days	204 ± 6	123 ± 13	125 ± 2	79 ± 4	107 ± 16	1 ± 11
		10 days	240 ± 54	167 ± 43	164 ± 41	76 ± 13	156 ± 42	−4 ± 3

^*a*^ Freshly prepared and frozen crude PM aliquots stored at −20°C for 4, 7, or 10 days, respectively.

^*b*^ OLI-S, oligomycin-sensitive. The OLI-S ATPase activities were measured and calculated (total minus oligomycin-insensitive ATPase activity) in the presence of 40 µM OLI.

^*c*^ VAN-S, vanadate-sensitive; VAN-IS, vanadate-insensitive. The VAN-S and VAN-IS ATPase activities were measured and calculated in the presence of 400 µM VAN.

^*d*^ The Cdr1-specific ATPase activity was determined by subtracting the corresponding mean OLI-S ATPase activity of the six AD control strains (ADs) from the OLI-S ATPase activity of the *CDR1* variant.

^*e*^ The Pma1-specific ATPase activity of the indicated strains was determined by subtracting their OLI-S ATPase activity from the VAN-S ATPase activity.

^*f*^ These are the mean values ± SDs of all six AD strains combined.

^*g*^ (−), no SD for this value because only one data point exists.

^*h*^ Cells were either harvested without starvation or starved for 1 h on ice prior to harvesting.

^*i*^ −, no Cdr1-specific ATPase activity.

### The ATPase activities of the Cdr1-G521 variants did not correlate with their compromised efflux capacity

**Fig 4:**
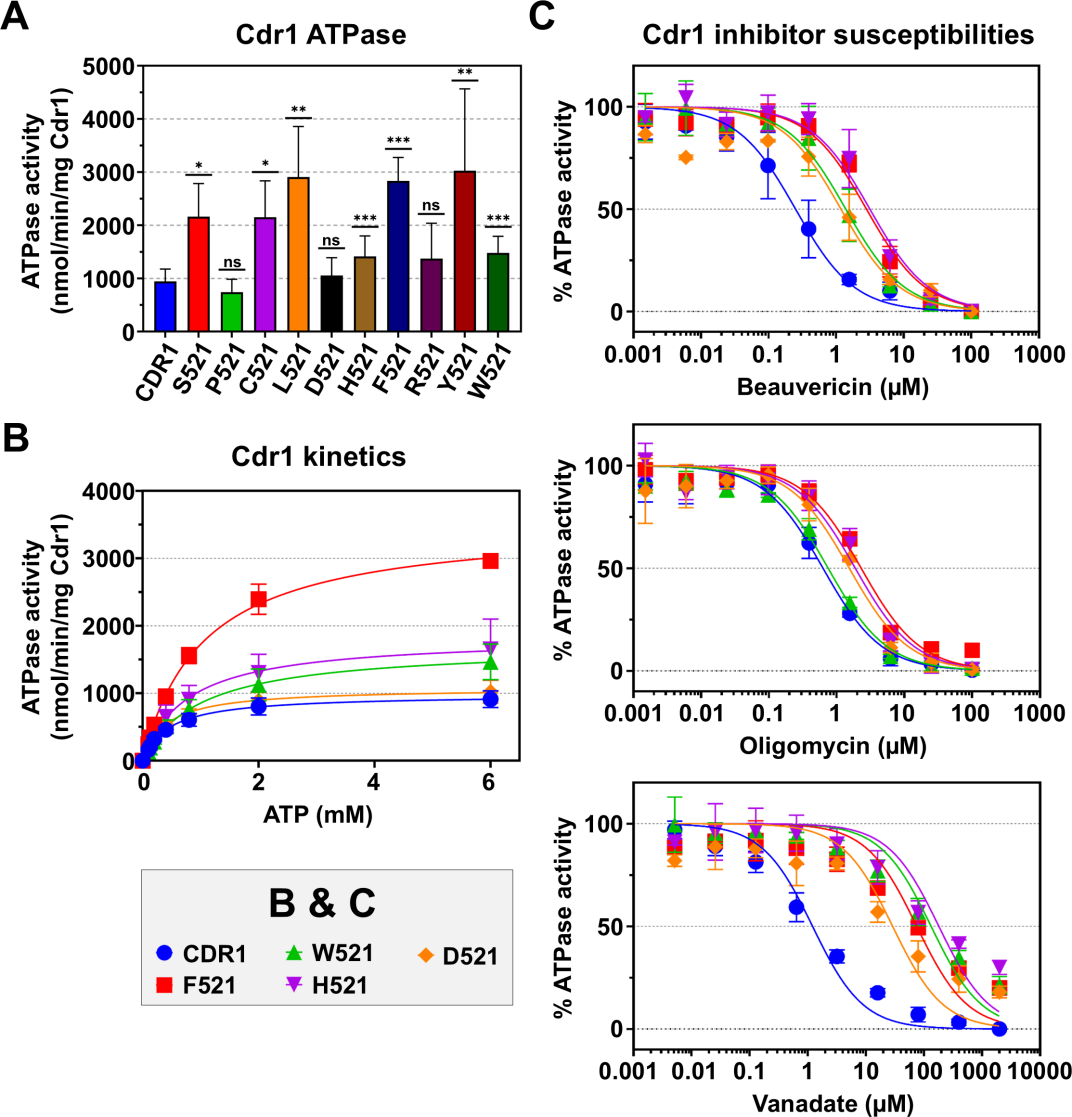
(**A**) Cdr1-specific ATPase activities of Cdr1 and Cdr1-G521 variants. The Cdr1 ATPase activities were calculated based on the assumption that Cdr1 accounted for 15% of the total crude PM protein of AD/CDR1B (determined from SDS-PAGE analysis). The data represent the means ± SD of at least three independent experiments using independently prepared PM samples. The Cdr1-specific ATPase activities were determined by subtracting the background ATPase activity of the negative control strain AD/pABC3 and accounting for the slightly different Cdr1 expression levels. Statistical significance analysis was performed between the 10 Cdr1-G521 variants and wild-type Cdr1 with Welch’s *t*-test (*P < 0.05, **P < 0.01, ***P < 0.001; ns, not significant). (**B**) Cdr1-specific ATPase kinetics of Cdr1 and F/H/W/D521 variants. Data represent the means ± SD of triplicate biological replicates of separately prepared PM samples. (**C**) Effects of Cdr1 efflux pump inhibitors on the Cdr1-specific ATPase activity of Cdr1 and F/H/W/D521 variants. Data represent the means ± SD of three independent experiments using independently prepared PM samples. The ATPase activities were normalized against the ATPase activity of their untreated controls, which were set to 100%. Dose-response curves and IC_50_ values were generated via nonlinear regression.

The ATPase activities of Cdr1 and Pdr5 are stimulated only minimally by substrate binding and remain constitutively active (43, 45). Variants with bulky residues (D/H/F/R/Y/W) exhibited ATPase activities comparable to, or exceeding, that of wild-type Cdr1 (940 nmol/min/mg Cdr1; Fig. 4A). Strikingly, L521, F521 and Y521 showed ~3-fold higher ATPase activities (2,900, 2,827 and 3,020 nmol/min/mg Cdr1), while H521 and W521 had ~1.5-fold-increased ATPase activities. R521 and D521 retained wild-type ATPase activity. Small residue substitutions also elevated the ATPase activity ~2- to 3-fold, except for P521, which had near-wild-type Cdr1 ATPase activity (Fig. 4A).

ATPase kinetics were investigated for wild-type Cdr1 and four large-residue variants (D/H/F/W521; Fig. 4B; Table 4). D521 showed wild-type-like parameters (*k*_cat_ = 3.0 s^−1^; *K_m_* = 0.4 mM), while F/W/H521 exhibited significantly increased catalytic turnover rates (*k*_cat_: 10, 4.8, and 5.2 s^−1^, respectively) and the *K_m_* values of F/W521 were also significantly increased (1.05 and 0.99 mM) (Table 4; Table S2). However, only F521 showed a marked gain in its catalytic efficiency (i.e., *k*_cat_/*K_m_* increased by ~60% to 9.5 × 10^3^ s^−1^ M^−1^) (Table 4). Despite impaired substrate transport, these mutants had enhanced ATPase activities, indicating that their transport defects likely result from spatial conflicts in the substrate entry pathway.

**TABLE 4:** Kinetic properties and inhibitor susceptibilities of the ATPase activities of Cdr1 mutants

Strain	% Cdr1 expression	Kinetic properties				IC_50_ (μM)		
		*V*_max_^*a*^ (nmol/min/mg Cdr1)	*K*_*m*_ (10^−3^ M)	*k*_cat_^*b*^ (s^−1^)	*k*_cat_/*K_m_* (10^3^ s^−1^ M^−1^)	BEA	OLI	VAN
AD/CDR1B	100	980	0.46	2.8	6.0	0.3	0.6	1.2
G521D	85	1,080	0.40	3.0	7.6	1.2	1.5	29
G521W	95	1,700	0.99	4.8	4.9	1.4	0.7	133
G521H	97	1,840	0.77	5.2	6.8	3.2	1.9	181
H521/C712S	53	800	0.37	2.2	6.1	2.1	2.2	274
H521/L1225I	99	1,587	0.63	4.5	7.1	3.5	2.9	15
G521F	94	3,533	1.05	10.0	9.5	2.8	2.3	75
F521/D511V	100	827	0.35	2.3	6.7	1.8	1.2	2.1
F521/A549V	100	3,880	0.86	11.0	12.8	3.5	10	7.3
F521/T658P	96	1,447	0.61	4.1	6.7	1.7	1.4	1.4
F521/G672R	42	1,093	0.30	3.1	10.3	4.8	40	0.6

^*a*^ Values account for the different Cdr1 expression levels and were calculated based on the assumption that Cdr1 accounted for 15% of the total crude PM protein in the wild-type strain (determined from SDS-PAGE analysis).

^*b*^ *k*_cat_ values of the Cdr1 ATPase activities are the number of ATP molecules hydrolyzed per second by one Cdr1 molecule.

### Large-residue substitutions at G521 abolished inhibition of the ATPase activity by various types of efflux pump inhibitors

We assessed the inhibitory effects of three Cdr1 inhibitors—beauvericin (BEA), OLI, and sodium orthovanadate (VAN)—on the ATPase activity of wild-type Cdr1 and the D/H/F/W521 variants. Excessively bulky or rigid inhibitors such as BEA and OLI (29, 46) can occupy most of the substrate-binding cavity (17), locking the protein in the inward-facing conformation, while VAN mimics phosphate (Pi) and traps the transporter in an outward-facing transition state by forming a stable ADP-VAN-Mg^2+^ complex at the catalytically active nucleotide-binding site 2 (NBS2) (47). All four G521 variants showed reduced inhibitor susceptibilities. BEA resistance increased ≥4-fold (half maximal inhibitory concentration, IC_50_: 1.2 μM–3.2 μM vs 0.3 μM for the wild-type) while OLI showed more moderate shifts from 0.6 μM to 0.7 μM–2.3 μM (Fig. 4C). Surprisingly, VAN inhibition was incomplete even at 2 mM in all mutants (Fig. 4C), with W/H521 IC_50_ values more than 100-fold those of wild-type Cdr1 (Table 4). These findings suggested that large G521 substitutions exert two mechanistic impacts: physically preventing inhibitors’ access to the efflux channel and disrupting the binding-release dynamics of ATP by disturbing NBD allostery.

### Secondary-site mutations in ITC- and R6G-resistant Cdr1-G521R/F/H/W/Y suppressor mutants cluster at important TMD contact regions

**Fig 5:**
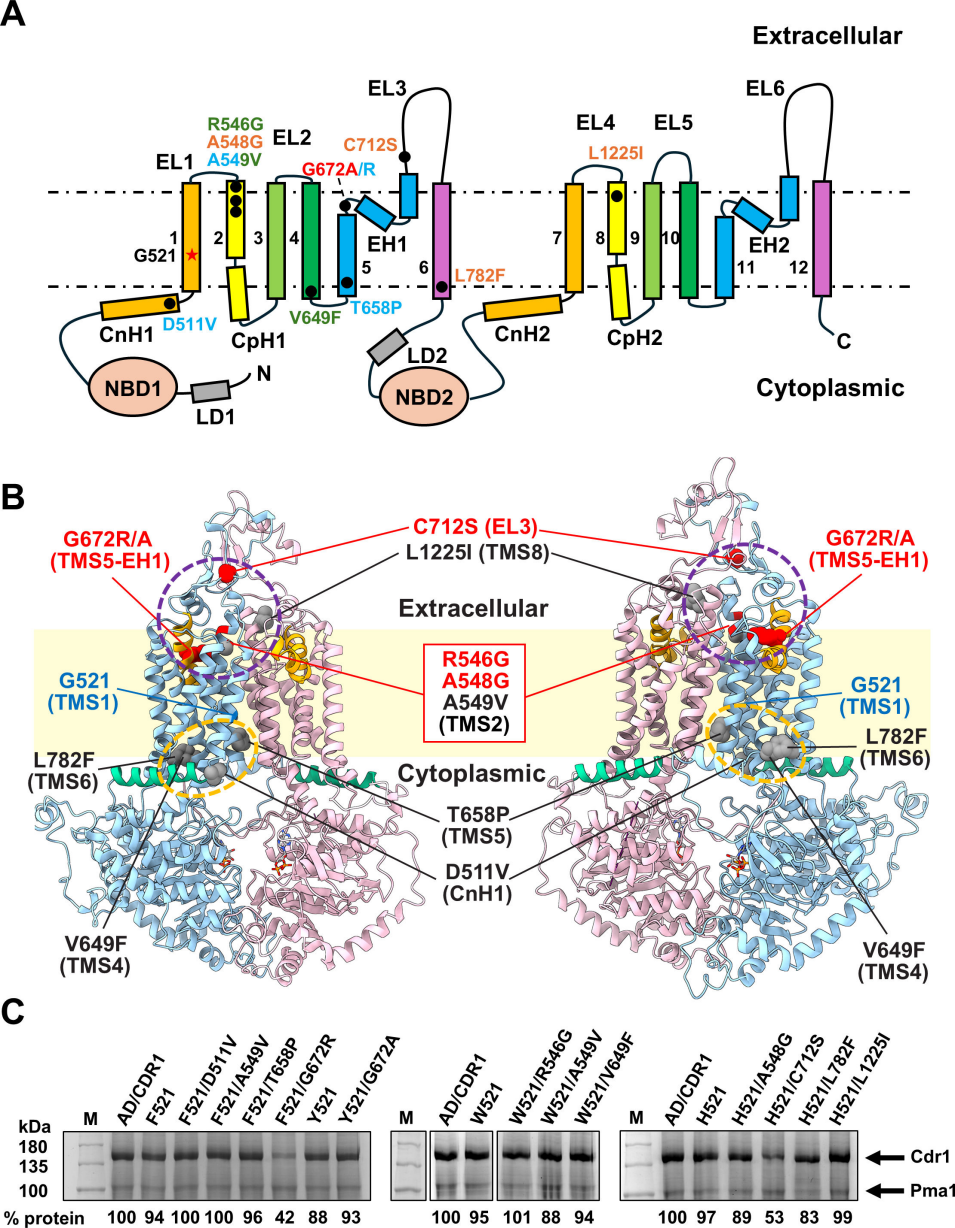
(**A**) Topological organization of Cdr1 highlighting F/H/W/Y521 suppressor mutation distributions. Secondary-site mutations of the ITC/R6G-resistant G521F/H/W/Y suppressor mutants are shown as black dots, with colored labels for F521 (blue), W521 (green), H521 (orange), and Y521 (red) suppressor mutations. The G521 residue (red star) and membrane-bilayer boundaries (dashed lines) are indicated. CnH, connecting helix; CpH, coupling helix; LD, linker domain; EH, elbow helix; EL, extracellular loop. (**B**) Cartoon models of the inward-facing (ATP/ADP-bound state) Cdr1 protein depicting G521 (blue) and all secondary-site mutations selected by R6G (red spheres) or ITC (gray spheres) in anterior (left) and posterior (right) views. TMD1 and NBD1 are in cyan, and TMD2 and NBD2 are in pink. EHs and CnHs are shown in orange and green, respectively. The membrane-bilayer boundaries are shown in pale yellow. Mutation clusters near the extracellular (violet) and cytoplasmic (orange) membrane boundary regions are indicated with dashed circles. (**C**) SDS-PAGE of 20 μg crude PM proteins of AD cells overexpressing Cdr1 (AD/CDR1B) or Cdr1-G521 variants and their suppressor mutants. Arrows indicate the 170 kDa Cdr1 and the 100 kDa Pma1 bands. M = MW markers. The % expression levels relative to Cdr1 are listed underneath the images.

Eighteen R/F/W/H/Y521 suppressor mutants were isolated under ITC (13) or R6G (5) selection pressure (Table 1; Fig. 5A). Six ITC suppressor mutants reverted the large R521 or W521 residues to smaller L/P/S(2)/C(2)521 residues, and four ITC suppressor mutants were part of a cytosolic membrane-bilayer boundary cluster (orange circle, Fig. 5B): D511V was a mutation of the helix break point between connecting helix 1 (CnH1) and TMS1, and V649F, T658P, and L782F were mutations at the bottom of TMS4, TMS5, and TMS6, respectively. The remaining three ITC suppressor mutants were near the top of TMS2 (A549V = 2) and TMS8 (L1225I) (Fig. 5). The five R6G suppressors were all near the center of the transporter (violet circle, Fig. 5B), at the top of TMS2 and TMS5 and in EL3: R546G and A548G were at the top of TMS2, G672R and G672A were both part of the conserved hydrophobic loop connecting TMS5 with elbow helix 1 (EH1), and C712S removed a disulfide bond just above TMS2 (Fig. 5B).

SDS-PAGE of crude PM preparations revealed comparable Cdr1 expression levels in wild-type, parental mutants, and most suppressor variants, except for F521/G672R and H521/C712S, the expression levels of which were reduced by ~50% (Fig. 5C).

### Substrate transport and ATPase activity of Cdr1-G521F/H/W/Y suppressor mutants

**Fig 6:**
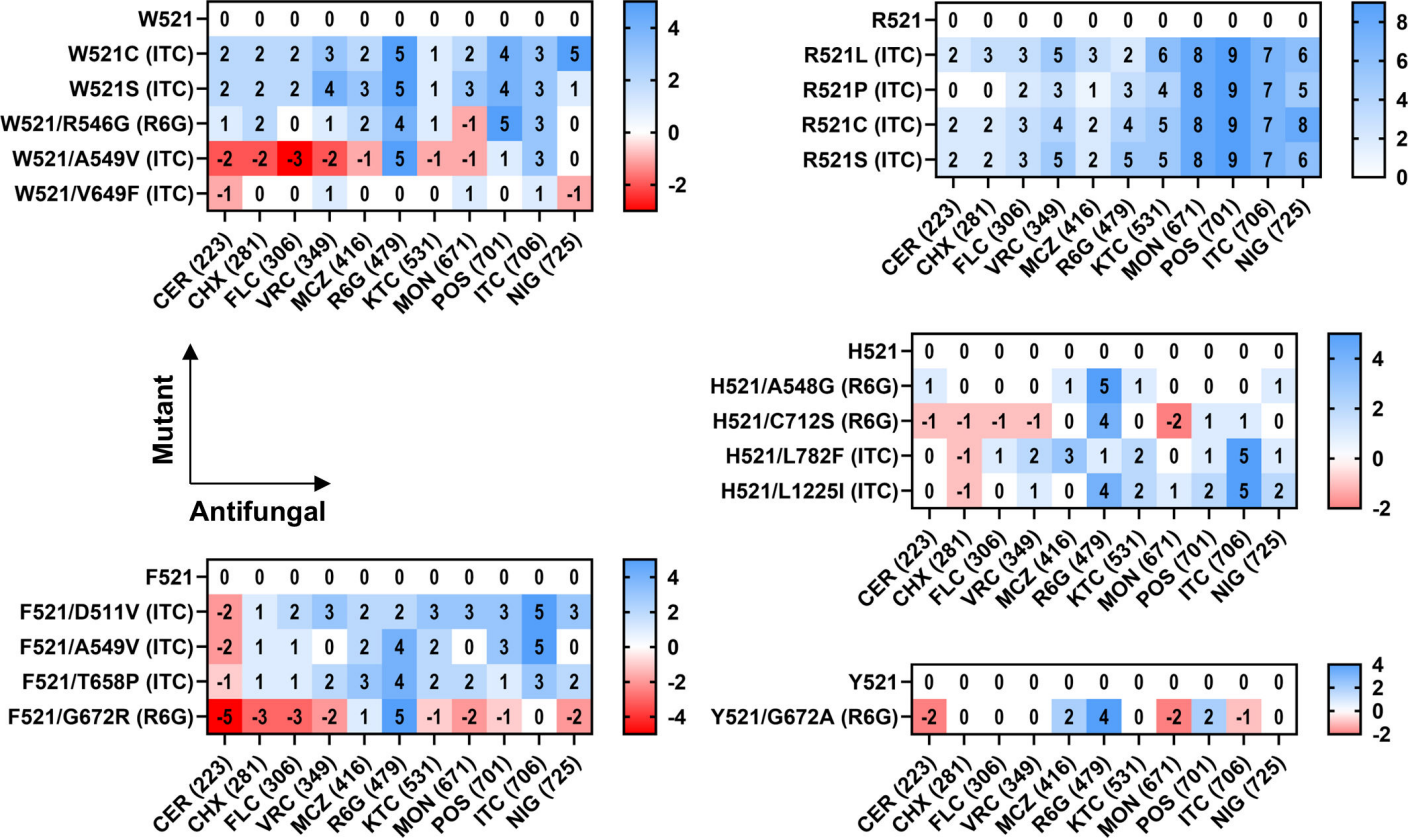
Heatmap of log_2_-transformed fold-increased (blue) or -decreased (red) MIC values of 11 antifungals for Cdr1-G521W, F, R, H, and Y variants and their respective suppressor mutants. Heatmap colors indicate fold changes relative to the parental mutant: blue for increased MIC values (0–9 = 1- to 512-fold) and red for decreased MIC values (0–5 = 1- to 32-fold). Antifungals (*x*-axis) are ordered by increasing MWs, listed in brackets. The antifungals that were used in the suppressor screenings are listed in brackets next to the *y*-axis.

As expected, the MICs for 11 antifungal compounds of the 18 suppressor mutants revealed enhanced resistance to the screening substrates (e.g., F521/D511V demonstrated >32-fold-increased ITC resistance compared to the parental mutant G521F; Fig. 6). Variants such as F521/D511V, F521/A549V, W521/R546G, and H521/L782F exhibited broad-spectrum resistance recovery, whereas others (F521/G672R, W521/A549V, and H521/C712S) displayed substrate-specific rescue (Fig. 6). R6G transport kinetic parameters corroborated MIC-based resistance profiles, with all suppressor mutants demonstrating significantly improved efflux capacity (Table 5; Fig. S5; Table S3).

**TABLE 5:** Kinetic properties of R6G transport and the Cdr1-specific ATPase activities of Cdr1-G521 variants and their respective “second-site suppressor” variants

Strain	% Cdr1 expression	R6G transport			ATPase activity (nmol/min/mg Cdr1)
		EQ (%RFU)	MAX (%ΔRFU/min)	t_1/2_ (min)	
AD/CDR1B	100	101 ± 2.5^*a*^	11 ± 0.5	5.5 ± 0.4	940 ± 227
G521F	94	4.5 ± 1.3	0.13 ± 0.01	21 ± 3.7	2,827 ± 427
F521/D511V	100	43 ± 2.3	2.2 ± 0.2	16 ± 1.6	760 ± 127
F521/A549V	100	80 ± 2.8	4.4 ± 0.2	12 ± 0.4	3,007 ± 467
F521/T658P	96	72 ± 7.7	5.3 ± 0.6	11 ± 0.6	1,373 ± 260
F521/G672R	42	169 ± 6.8	12	8.7 ± 0.4	1,167 ± 353
G521H	97	67 ± 1.6	3.5	14 ± 1.9	1,407 ± 373
H521/A548G	89	113 ± 6.1	11	7.0 ± 1.1	987 ± 140
H521/C712S	53	171 ± 3.6	14	6.3 ± 0.7	813 ± 200
H521/L782F	83	114 ± 3.3	6.3	14 ± 0.6	1,180 ± 400
H521/L1225I	99	81 ± 15	5.8	6.7 ± 0.6	1,293 ± 220
G521W	95	11 ± 5.0	0.6	20 ± 2.4	1,473 ± 300
W521/R546G	101	77 ± 11	6.2	9.5 ± 0.3	1,180 ± 193
W521/A549V	88	107 ± 0.8	9.0	10 ± 0.7	760 ± 160
W521/V649F	94	22 ± 5.0	1.1	17 ± 0.6	2,300 ± 380
G521Y	88	–^*b*^	–	–	3,020 ± 1,440
Y521/G672A	93	89 ± 1.3	8.8	4.7 ± 0.07	980 ± 300

^*a*^ Values are the means of two and three and up to 27 independent experiments (±SDs) for the R6G transport and the ATPase activity, respectively, accounting for the different Cdr1 expression levels.

^*b*^ –, no detectable R6G transport.

**Fig 7:**
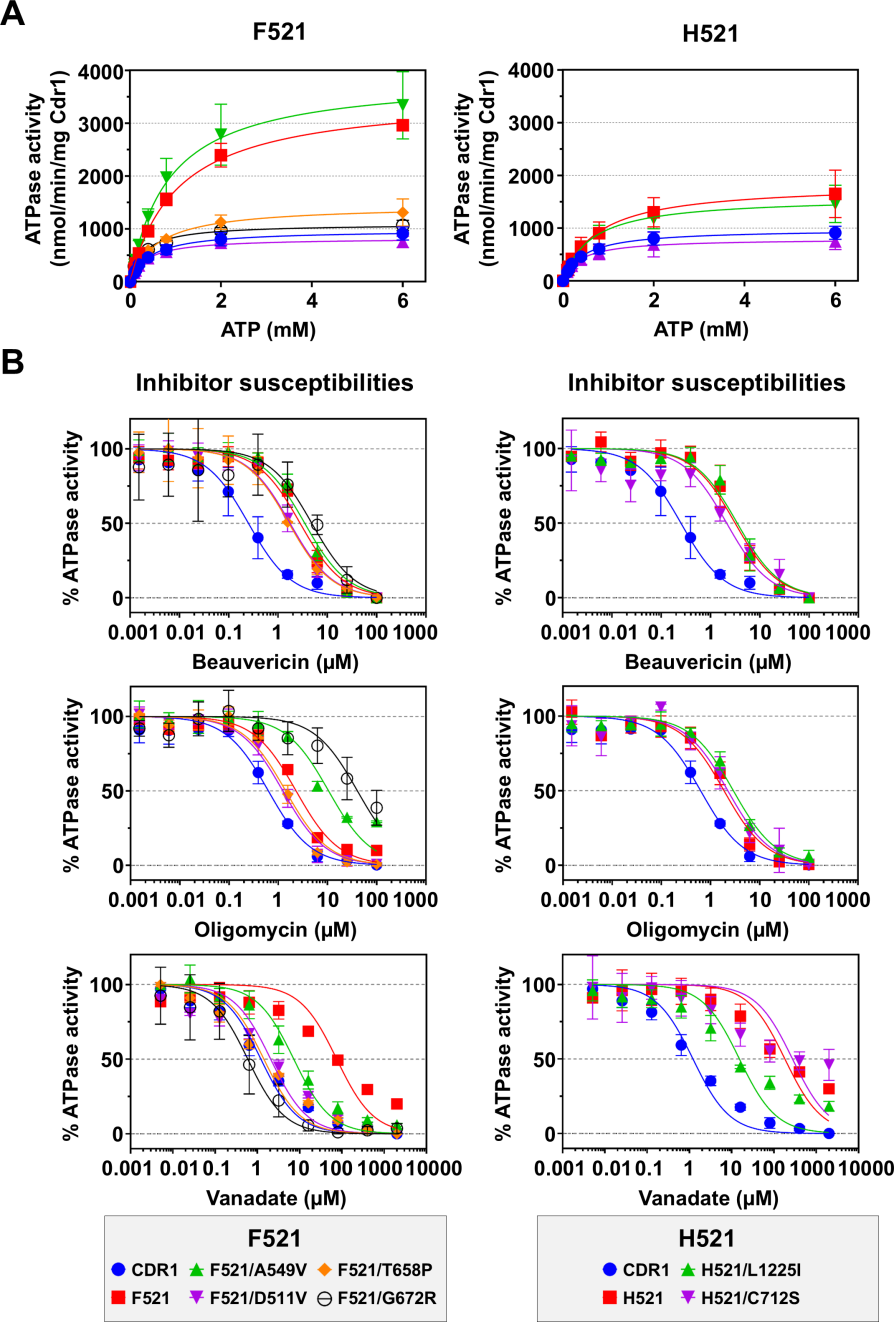
(**A**) Cdr1-specific ATPase kinetics were determined for wild-type Cdr1, G521F, G521H, and their suppressor mutants. Data represent the means ± SD of three biological replicates from separately prepared PM samples. (**B**) Effect of three efflux pump inhibitors on the Cdr1-specific ATPase activity of wild-type Cdr1, G521F, G521H, and their suppressor mutants. Data are the means ± SD of three biological replicates of separately prepared PM samples. The Cdr1-specific ATPase activities were normalized to the untreated control samples, which were set to 100%. Dose-response curves and IC_50_ values were generated via nonlinear regression.

Almost all suppressor mutants fully or partially recovered wild-type Cdr1 ATPase activities (760–1,373 nmol/min/mg Cdr1; Table 5; Fig. 7A). The only exceptions were the H521/L1225I, F521/A549V (1,293 and 3,007 nmol/min/mg Cdr1) and the W521/V649F (2,300 nmol/min/mg Cdr1) variants that had very similar or even ~1.6 times higher ATPase activities than their parent strains G521H, G521F, and G521W (1,407, 2,827, and 1,473 nmol/min/mg Cdr1; Table 5; Fig. 7A). Although all four G521F (i.e., D511V, A549V, T658P, G672R) and both G521H (i.e., C712S, L1225I) suppressor mutants that were tested had lower *K_m_* values than their parent strains (Table 4), the reductions for only three of them (D511V, T658P, G672R) were statistically significant (Table S2).

The acquired secondary-site mutations differentially modulated the inhibitor responsiveness of the ATPase activity (Table 4; Fig. 7B). While most of these G521F and G521H suppressor mutants maintained almost parental-level resistance to BEA (IC_50_ 1.7 μM–4.8 μM) and OLI (IC_50_ 1.2 μM–2.9 μM) (Table 4), two variants exhibited profound OLI resistance: F521/G672R (40 μM) and F521/A549V (10 μM), with even 100 μM OLI failing to achieve complete ATPase inhibition (residual activity >30%; Fig. 7B). Conversely, wild-type Cdr1 VAN sensitivities (1.2 µM) were almost completely restored in all G521F suppressor mutants and in H521/L1225I (0.6 μM–15 μM). The only exception was the H521/C712S suppressor mutant, the dramatically increased VAN resistance (274 µM) of which remained largely unchanged from the G521H parental mutant (181 µM; Table 4; Fig. 7B).

## DISCUSSION

Models for the transport cycle of asymmetric PDR transporters (29, 48) propose that one ATP molecule is bound at all times to the non-catalytic NBS1. After substrate entry, the exchange of ATP for ADP at the catalytically active NBS2 right underneath the entry gate triggers rigid NBD body motions that close the entry gate and force substrate release through a hydrophobic exit valve (48). Subsequent ATP hydrolysis and the release of Pi return the transporter to the inward-facing conformation.

Studies of human ABCG transporters proposed that both the size and shape of the substrate-binding pocket (49), as well as the conformational flexibility provided by polar and hydroxylated residues (50), influence substrate selectivity. A study of the entire *Candida krusei* (recently renamed *Pichia kudriavzevii*) PDR transporter family provided further insights into the substrate selectivity of PDR transporters. While Abc11 and Abc12 have evolved to transport larger and smaller compounds, respectively, Abc1 is the most efficient multidrug efflux pump of *C. krusei* (51). Others have proposed that *S. cerevisiae* Pdr5 preferentially transports larger substrates because they have more interactions with the substrate-binding cavity (52). Like Pdr5, *C. albicans* Cdr1 possesses a remarkably broad substrate specificity, ranging from antifungal agents to human steroid hormones and phospholipids (53, 54), and it too transports larger substrates more efficiently (29). This size-dependent transport phenotype was reversed in the Cdr1-G521R variant (29).

### G521 is a key gating residue but also an important TMD contact residue of Cdr1

**Fig 8:**
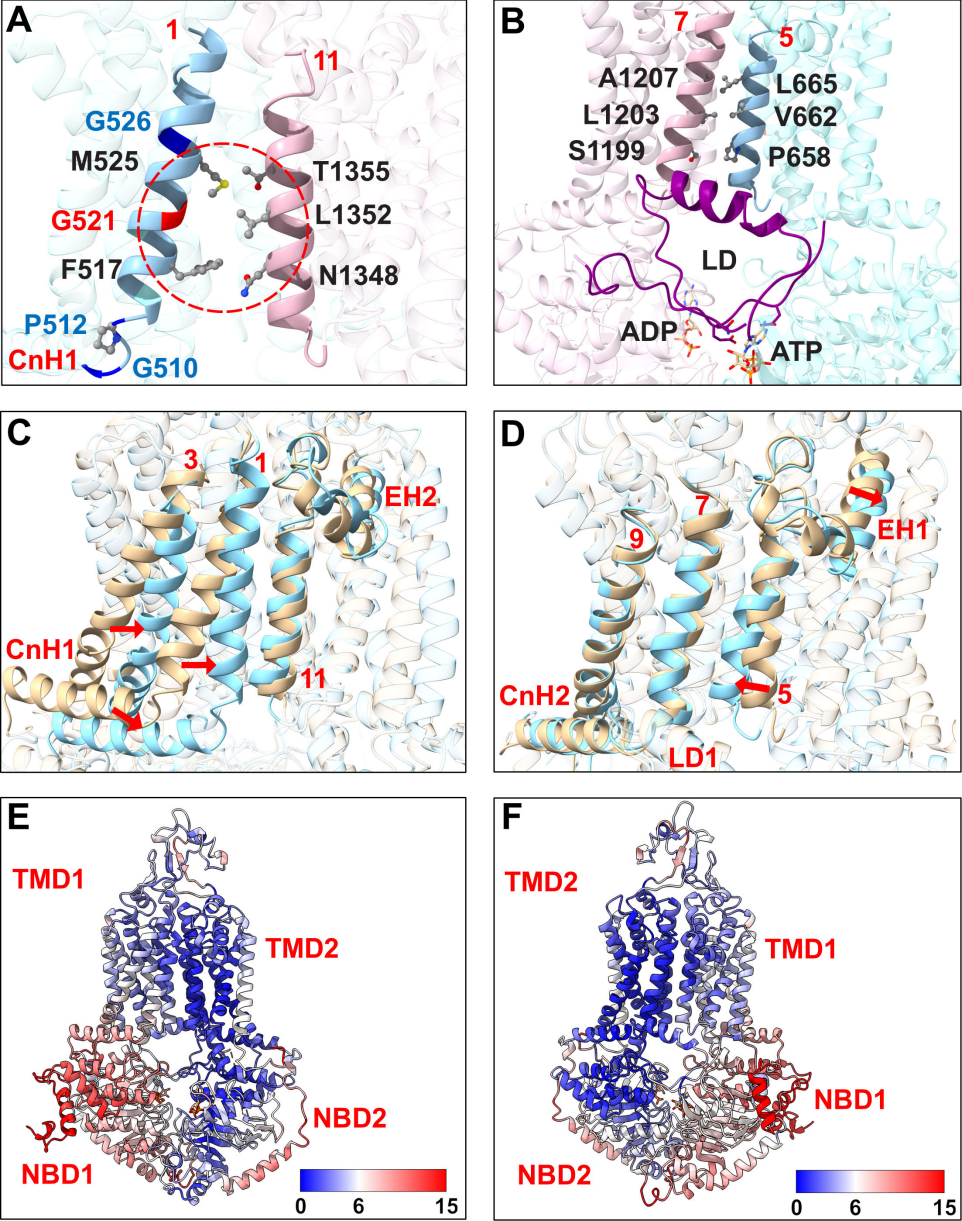
Structural features of the substrate entry gate (**A**) at the front and a comparison with the equivalent region at the rear of the transporter (**B**) in the inward-facing (ATP/ADP-bound) conformation of Cdr1. G521 is in red, and other residues that provide flexibility for TMS1 are in blue. The gate-surrounding TMS1 and TMS11 residues and contact residues between TMS5 and TMS7 at the equivalent region at the rear of Cdr1 are shown in ball-and-stick representation (O, red; C, gray; N, blue; S, yellow; Hs are omitted). TMD1, TMD2, and linker domains are colored in cyan, pink, and purple, respectively. (**C, D**) Structural alignments of the inward- (yellow) and the outward-facing (cyan) conformations of Cdr1, highlighting the significantly greater movement of the gating region (i.e., CnH1, TMS1, TMS3) at the front (**C**) than of the equivalent region at the rear (**D**), which remains closed throughout the transport cycle. Red arrows indicate the directional movement of TMSs during the conformational transition. (**E, F**) Correlation surface mapping of structural deviations between the inward- and the outward-facing conformations, with a color gradient representing Cα RMSD values ranging from 0 to 15 Å (blue to red). Panel **E** and **F** images are front and rear views, respectively.

The lack of a side chain in G521 and the deviation of TMS1 from the vertical plane create an opening between TMS1 and TMS11 (Fig. 8A) for substrates to enter the transporter. Conserved glycine and proline residues at the bottom (G510, P512) and near the top (G526) of TMS1, and G521 at its center (Fig. 8A), possibly provide TMS1 with the necessary flexibility to allow a variety of different substrates to enter, akin to *Cyanidioschyzon merolae* ABCB1, with conserved glycine and proline residues in TMS4 that regulate entry of substrates between TMS4 and TMS6 (55). The PDR transporter-specific linker domain (purple; Fig. 8B) (21) promotes constant binding of ATP to NBS1 and ensures that TMS5 and TMS7 are closed at all times during the transport cycle (Fig. 8C and D). The asymmetric nature of Cdr1 results in significant movements of NBD1 and TMD1 during the transport cycle, while the C-terminal half acts as a rigid scaffold (Fig. 8E and F). ATP binding at the NBS2 causes TMS1 to move markedly closer to TMS11 to seal the entry pathway—a motion absent on the opposite side of the transporter (Fig. 8C and D).

Our homology model-based predictions are supported by the experimental cryo-EM structure of Cdr1 that became available during the course of our work (20). In this structure, the G521 residue is located precisely at the center of TMS1 and lines a solvent-accessible entry gate that forms between TMS1 and TMS11 (Fig. 8A). Absence of a side chain in the wild-type glycine residue creates a crucial opening that is predicted to control substrate access to the central binding cavity. Our findings, which show that replacing glycine with bulkier residues progressively restricts the transport of larger substrates, are therefore in agreement with the experimentally determined architecture.

The detailed characterization of the G521 variants supports a role for G521 as the gatekeeper of Cdr1. The fact that leucine and aspartate substitutions of G521, despite their similar size, affect the transport of large substrates quite differently implies that the negatively charged D521 residue impairs entry and/or efflux of larger compounds much more efficiently than L521 (Fig. 2B). The proline substitution of P521 was another notable exception. There was no enhancement in the transport of smaller compounds and minimal impact on the transport of larger substrates, possibly because it provides similar flexibility to TMS1 as G521. All other G521 substitutions caused a significant shift in the substrate preference toward smaller substrates, while the transport of larger substrates was more severely impaired with increasing side-chain bulk (Fig. S3).

Interestingly, G521 substitutions also influenced ATP hydrolysis. Similar effects on the TMD-NBD interdomain communication were previously reported for mutations of key TMD contact residues of Cdr1, Pdr5, and ABCB1 (35, 56, 57). This suggests that the G521 substitutions also affected local rearrangements at the catalytically active NBS2 just underneath the entry gate, which accelerated the release of Pi, reduced the likelihood of VAN trapping, and dramatically increased the ATPase activity. However, these effects were largely independent of the size-dependent substrate transport phenotypes described above.

### Secondary-site mutations reveal key TMD contact residues involved in proper gating and the TMD-NBD interdomain communication of Cdr1

Among the 10 secondary sites that, when mutated, could restore the severely impaired R6G or ITC transport of the G521 variants, mutations in 8 have previously been characterized in Cdr1 and/or Pdr5 (Table 6). There was a clear difference in the types of mutations that could recover ITC or R6G transport.

**TABLE 6:** Previous studies of Pdr5 and Cdr1 investigating the effects of mutations of the secondary mutation sites identified in the Cdr1-G521F/H/W/Y suppressor mutants

Cdr1	Pdr5	Region	Cons.^*a*^	Phenotype in previous studies	References
R546	R556	TMS2	R	Cdr1-R546A: could transport most of the test drugs Cdr1-R546T: FK506 resistant, 50% reduced ATPase activity	(27, 58)
A548	S558	TMS2	GAS	Cdr1-A548G: no KTC transport, wild-type expression levels and ATPase activity	(58)
				Pdr5-S558Y: no CHX transport, no inhibition of the ATPase activity by clotrimazole, wild-type expression levels but 50% reduced ATPase activity Pdr5-S558A: no impact on Pdr5 expression or function	(57)
A549	A559	TMS2	AVS	Cdr1-A549G: no transport of most of the test drugs, wild-type expression levels and ATPase activity	(58)
V649	L659	TMS4	VAL	Cdr1-V649A: no KTC transport, wild-type expression levels and ATPase activity	(59)
T658	V668	TMS5	TV	Cdr1-T658A: no impact on Cdr1 transport function	(58)
G672	G682	TMS5	G	Cdr1-G672A: no transport of most of the test drugs, wild-type expression levels, and ATPase activity Pdr5-G682A: misfolded and mislocalized	(60) (48)
C712	C722	EL3	C	Cdr1-C712S: FK506 resistant, 50% reduced ATPase activity Cdr1-C712A/C732S: wild-type expression levels, partially impaired FLC transport and ATPase activity	(27, 36)
L782	L792	TMS6	LF	Cdr1-L782A: no transport of certain drugs, wild-type expression levels and ATPase activity	(58)

^*a*^ Amino acid conservation in these regions based on the alignment of 42 cluster A PDR transporters (13).

**Fig 9:**
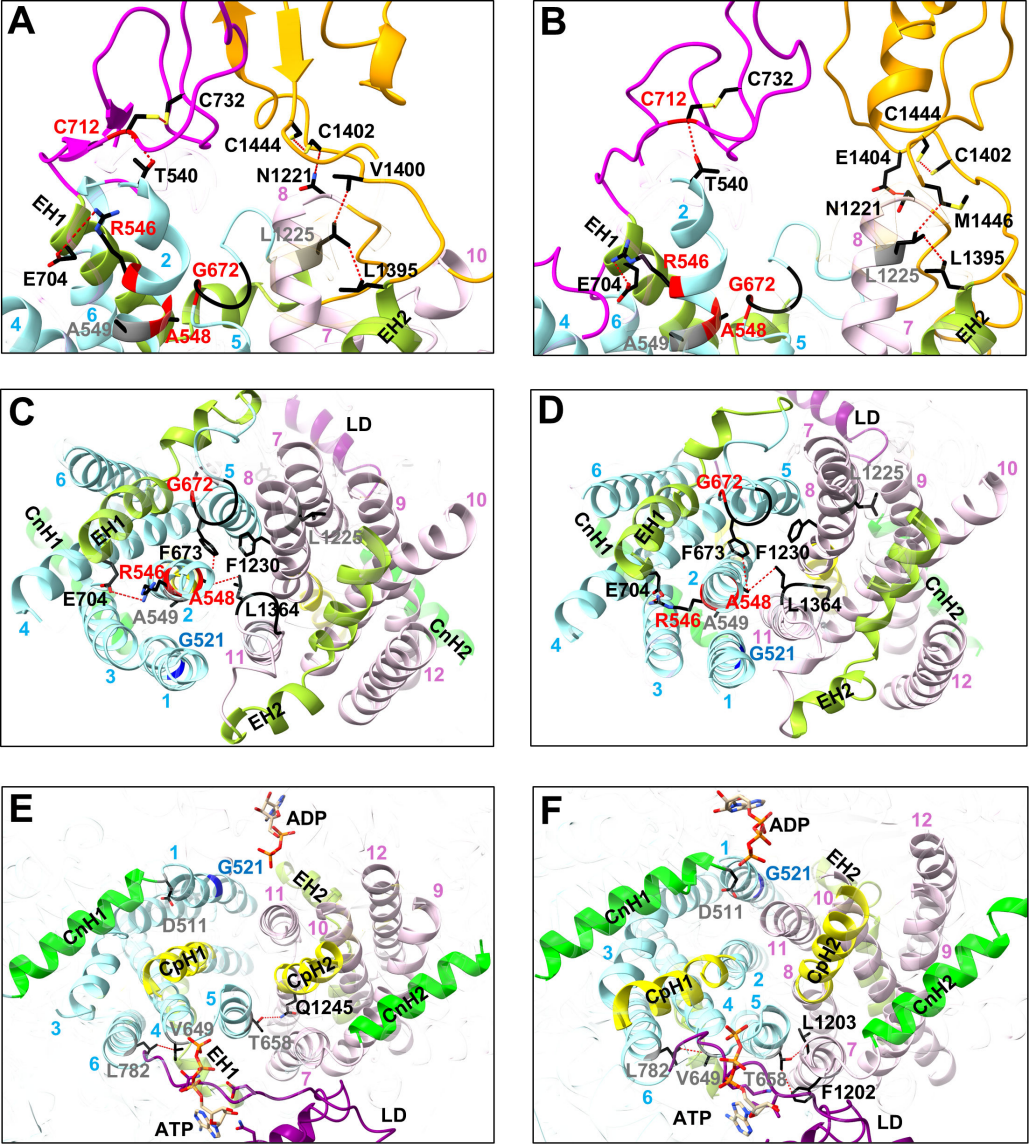
Cartoon models of the inward- (**A, C, E**) and the outward-facing (**B, D, F**) conformations of Cdr1 illustrating potential interactions involving the 10 secondary-site suppressor mutations of the G521W, F, H, and Y variants. Panels **A, B, C, D, E** and **F** highlight the EL-TMD boundary (**A, B**), the centrally located hydrophobic valve (**C, D**), and the bottom of the TMD (**E, F**), viewed from the top, front, and underneath of Cdr1, respectively. For clarity of view, structures in front of the highlighted zones are transparent. Side chains of important residues are shown as sticks (O, red; C, black; N, blue; S, yellow; Hs are omitted). The backbones of G521 and R6G- and ITC-isolated mutant residues are shown in blue, red, and gray, respectively. The hydrophobic exit valve (black sticks) in panels **C, D** is formed by F673 and L1364, members of two conserved hydrophobic loops (black cartoon) that connect TMS5 with EH1 and TMS11 with EH2, respectively; A548 (TMS2) and F1230 (TMS8; black sticks) complete the hydrophobic plug. The disulfide bonds between C712–C732 of EL3 and C1402–C1444 of EL6, and contacts between A548 (TMS2) and hydrophobic valve residues, and R546 (TMS2)–E704 (EH1), T540 (TMS2)–C712 (EL3), L1225 (TMS8)–L1395 (EH2), L1225–V1400 (EL6), N1221 (TMS8)–C1402, L1225–M1446 (EL6), N1221–E1404 (EL6), V649 (TMS4)–L782 (TMS6), T658 (TMS6)–Q1245 (CpH2), T658–F1202 (TMS7), and T658–L1203 (TMS7) are shown as red dashed lines. TMD1: cyan; TMD2: pink; EHs: yellow-green; CnHs: bright green; LD: purple; CpHs: yellow; EL1/2/3: magenta; EL4/5/6: orange.

The four R6G “suppressor sites” clustered in the upper TMD1 region (Fig. 9A through D), three of which (R546G, A548G, C712S) were previously identified as Cdr1 FK506 resistance hotspot 1 residues (27). The fourth residue G672 (giving rise to G672R and G672A) is part of a conserved loop (GFVI; black, Fig. 9) that connects TMS5 with EH1 and which is part of the hydrophobic exit valve (Fig. 9) (48). A548, at the top of TMS2, is in direct contact with the hydrophobic exit valve residues F673 (TMS5) and L1364 (TMS11; Fig. 9C and D), the Pdr5 equivalent residues of which, F684 and M1373, were part of the FK506 resistance hotspot 2 (27). The conserved R546 residue connects TMS2 to the conserved E704 residue at the C-terminus of EH1 (Fig. 9C and D), and C712 forms a disulfide bond with C732, which is in direct contact with T540 of EL1, right above TMS2 (Fig. 9A and B). The A548G, G672A/R, and C712S mutations quite specifically recovered the R6G efflux of G521H, F, and Y variants, while the R546G mutation recovered the transport capacity of G521W for most test substrates (Fig. 6). It appears that the G521 variants had not only impaired entry of larger substrates, including R6G, but they were also quite significantly impaired in R6G efflux which required an adjustment of the exit valve architecture to recover R6G efflux and/or prevent R6G reflux, as reported for the Pdr5-F684L hydrophobic valve mutant (48).

The six ITC “suppressor sites” also clustered near the top of the hydrophobic exit valve (A549V and L1225I; Fig. 9A through D) and they also included mutations at important TMS contact regions (D511V, V649F, T658P, L782F) near the bottom of TMD1 (Fig. 9E and F). D511 connects TMS1 with CnH1, V649 and L782 are in close contact with each other at the bottom of TMS4 and TMS6, respectively, and T658, at the bottom of TMS5, is in close contact with TMS7 and TMS8 residues (Fig. 9E and F). A549 is in close contact with TMS1 and TMS3 (Fig. 9C and D), and L1225 is the R546 equivalent residue that connects TMS8 with L1395 at the C-terminus of EH2 (Fig. 9C) and is also in close contact with EL6 residues near the disulfide bond 1-equivalent disulfide bond 3 region between C1402 and C1444, right above TMS8 (Fig. 9A and B). Apart from W521/A549V, which also quite specifically recovered R6G and ITC transport and negatively affected the transport of smaller compounds, F521/A549V and the remaining five ITC “suppressor site” mutations recovered the transport of most test substrates (Fig. 6), indicating an adjustment to the entry gate architecture for most ITC suppressors.

In summary, this study provides the first comprehensive analysis of the substrate entry gate of any type V ABC transporter. We identified G521 at the center of TMS1 as the key *C. albicans* Cdr1 gating residue that regulates access of compounds to the substrate-binding pocket and serves as a pivotal structural element that translates rigid-body motions between the NBDs into the large conformational changes of the TMDs that drive substrate translocation through a hydrophobic exit valve at the top of the transporter. The substrate preferences of other PDR transporters may be regulated in a similar fashion by adjusting the gate architecture at their equivalent TMS1 regions. Our findings offer new insights into the functional mechanism of fungal PDR transporters and establish a foundation for the rational design of selective Cdr1 efflux pump inhibitors.

## MATERIALS AND METHODS

### Strains and chemicals

The *CDR1* A or B allele of *C. albicans* 10261 (61) was overexpressed from the genomic *PDR5* locus of *S. cerevisiae* AD1-8u^-^ (39), ADΔ (30), or ADΔΔ (40). Cdr1-expressing strains and empty-transformation-cassette-containing negative control strains were generated via transformation of host strains with a *CDR1* expression cassette (from pABC3-CDR1A or pABC3-CDR1B) or an empty vector fragment, as described previously (30). Large-residue G521 mutations (G521D, H, F, Y, W) were introduced using PCR-based site-directed mutagenesis (36) with DNA oligomer primers listed in Table S4. Natural suppressor variants of G521 mutants (G521R, H, F, Y, W) were isolated by plating 1 × 10^6^–1 × 10^7^ yeast cells onto complete supplement mixture (CSM) agar plates (0.077% CSM [Bio 101, Vista, CA], 0.67% yeast nitrogen base without amino acids [Difco], 2% glucose, 2% agar) supplemented with high concentrations of ITC or R6G (two to four times the MIC), followed by 1 to 2 week incubation at 30°C. All yeast strains used in this study are listed in Table 1. Yeast cells used for PM preparation were cultured in YPD medium (1% yeast extract, 2% peptone, 2% glucose; Difco) at 30°C.

The following chemicals were purchased: MON, OLI, VAN, BEA, and 2-Deoxy-D-glucose (2-DOG) from Sigma-Aldrich; MCZ, NIG, CER, and R6G from FUJIFILM Wako Pure Chemical Corporation; POS and ITC from Tokyo Chemical Industry; VRC and CHX from NACALAI TESQUE; FLC and KTC from LKT Laboratories.

### Determination of antifungal susceptibilities

Antifungal MICs were measured by a twofold serial dilution in CSM medium (pH 7.0) as described previously (36) and defined as the lowest compound concentration reducing growth by at least 90%.

### Whole-cell R6G efflux assay

Yeast cells were cultured in 15 mL CSM medium at 30°C from an optical density at 600 nm (OD_600_) of 0.001 to 1.0, and then incubated with 20 μM R6G and 5 mM 2-DOG in 5 mL of 50 mM HEPES buffer (pH 7.0) for 90 min at 30°C. After two washes with cold HEPES buffer, cells were resuspended in 1 mL HEPES (pH 7.0), and 100 μL (~1 × 10^7^ cells) was added to each well of a 96-well plate in triplicate for two experimental groups. Efflux was initiated by adding 100 μL of 40 mM glucose in HEPES to the treatment group, while 100 μL of HEPES without glucose was added to the control group. Extracellular R6G fluorescence (Ex: 525 nm, Em: 550 nm) was measured with a microtiter plate reader. Background fluorescence from control wells was subtracted from the corresponding experimental readings. For kinetic analysis, cells were incubated with varying R6G concentrations (5 μM–30 μM).

### Isolation of crude PMs

Yeast cells were cultured, harvested, and stored following a modified version of a previously described protocol (36), in which 10 mL of preculture was used to inoculate 100 mL of YPD medium at an initial OD_600_ of 0.001, followed by incubation at 30°C until mid-logarithmic growth phase (~OD_600_ 1.0). PMs were isolated using a modified bead-beating protocol (36). Forty ODU (1 ODU = 1 mL cell culture of an OD_600_ of 1) of the mid-logarithmic cells were harvested, and the cell pellet was washed once with 100 mL ice-cold water. The cells were harvested once more, resuspended in 40 mL ice-cold water, and starved for 1 h before harvesting and resuspending the cell pellet in 500 µL homogenizing buffer (HB; 50 mM Tris-Cl, pH 7.5; 2 mM EDTA; 20% [vol/vol] glycerol) and storing the cell suspension at −20°C. For the isolation of crude PMs, the 40 ODUs of frozen cell stocks were defrosted and supplemented with 5 µL 100 mM PMSF before disrupting the cells with 400 µL of 0.5 mm silica beads in 2.0 mL microcentrifuge tubes using a 1600 MiniG Cell Lyser at 1,300 rpm for 4 min in a pre-chilled (−20°C) lysis container. Using a Cell Lyser rather than breaking cells manually with a vortex mixer resulted in more consistent yields (~0.2 mg crude PM protein) and highly reproducible Cdr1-specific ATPase activities of ~150–200 nmol/min/mg of fresh crude PM protein. The amount of PM protein was determined using the Lowry-based DC protein assay kit (Bio-Rad). Cdr1/Pma1/50 kDa protein expression levels, and their proportions in PM proteins, were assessed by SDS-PAGE using 7% acrylamide gels loaded with 20-30 µg of PM proteins, stained with Coomassie brilliant blue R250 and quantified with ImageJ (62).

### ATPase assay

The ATPase activity of crude PM aliquots (at least three ~70 µL aliquots per PM sample) stored frozen at −20°C for no longer than 7 days was measured in duplicate in 96-well plates using a colorimetric molybdate-based assay. Each well received 55 μL of prewarmed ATPase assay cocktail (109.1 mM Tris-Cl, pH 7.5; 109.1 mM KNO₃; 0.44 mM ammonium molybdate; 10.9 mM sodium azide) and 5 μL of PM proteins (3–10 μg) or phosphate standards (0–100 nmol Pi), followed by the addition of 60 μL of 12 mM prewarmed Mg-ATP to initiate the reaction. After 1 h incubation at 30°C, the reaction was stopped by adding 130 μL of development reagent (1.6% [wt/wt] sodium L-ascorbate, 1% SDS, 1.2% ammonium molybdate in 0.6 M H_2_SO_4_). Absorbance at 750 nm was measured after 10 min incubation at RT, and Pi release was quantified using a standard curve. OLI-IS and VAN-IS ATPase activities of six AD strains and ADΔΔ strains expressing either the wild-type Cdr1 or an E1027Q variant were measured in the presence of 40 µM OLI and 400 µM VAN. Cdr1-specific activity was determined by subtracting total background values from the negative control strain AD/pABC3. Kinetic parameters (*K_m_*, *V*_max_) were determined by fitting ATP dose-response curves (0 mM–6 mM) to a Michaelis-Menten model. Inhibitor effects (BEA, OLI, VAN) were assessed over a concentration range of 0 μM–100 μM (VAN: up to 2,000 μM), and IC_50_ values were calculated by nonlinear regression analysis.

### Homology modeling of Cdr1 and Cdr1-G521R

Homology models of the apo, inward-open with ATP/ADP, and outward-open conformations of *C. albicans* Cdr1 (UniProt accession code Q5ANA3) were generated using the RosettaCM hybridize protocol (63). The cryo-EM structures of *S. cerevisiae* Pdr5 (PDB IDs: 7P05 and 7P06) served as structural templates. The protocol utilized 3-mer and 9-mer fragment libraries generated with the Rosetta fragment_picker from secondary structure predictions by PSIPRED (64) and RaptorX (65). For each state, an ensemble of 20 decoys was generated and subjected to a constrained, full-atom relaxation, and the lowest-scoring model was selected for further refinement. This refinement entailed removing the unstructured N-terminus and a flexible internal loop (residues 817–860), manually placing co-factors to align with template poses, and performing a final local energy minimization. The stereochemical quality of the final models was validated using MolProbity (66), yielding excellent scores (0.89–1.37). Modeller 10.5 (67) was used to generate the homology models for CaCDR1A-G521R using either the inward-facing (PDB ID: 7P05) or the outward-facing (PDB ID: 7P06) conformations of *S. cerevisiae* Pdr5 as templates. Sequence alignments were generated using the T-Coffee server (68).

### Statistical analyses and model visualization

Statistical analyses were performed with GraphPad Prism (GraphPad Software Inc.) or using the SciPy library (v.1.15.2) in Python (v.3.11). ATPase activity and whole-cell R6G efflux data were analyzed with the F-test to compare variances, followed by an unpaired *t*-test with Welch’s correction. The extra sum-of-squares F-test was used to evaluate statistically significant differences between the ATPase kinetic properties and the IC_50_ values of the Cdr1 variants. All structural visualizations were performed using UCSF ChimeraX (69).

## SUPPLEMENTAL MATERIAL

File S1 mbio.03746-25-s0001.docx In-depth description of additional data.

Supplemental material mbio.03746-25-s0002.docx Supplemental tables and figures.

ASM does not own the copyrights to Supplemental Material that may be linked to, or accessed through, an article. The authors have granted ASM a non-exclusive, world-wide license to publish the Supplemental Material files. Please contact the corresponding author directly for reuse.
