# Supplementary material for: G521 is the gatekeeper and a key transmembrane domain contact residue of Candida albicans Cdr1
Source: mBio. 2026 Feb 26;17(4):e03746-25. doi: 10.1128/mbio.03746-25 (PMC13059742; doi:10.1128/mbio.03746-25)
Supplement: File S1 — In-depth description of additional data. [file mbio.03746-25-s0001.docx]

**Supplementary File S1**

**Characterization of crude plasma membranes isolated from various yeast strains using the optimized small-scale plasma membrane isolation protocol.**

In order to identify the most reliable method to determine the Cdr1-specific ATPase activity of crude plasma membrane (PM) preparations, crude PM samples were isolated from the eight *Saccharomyces cerevisiae* strains listed in **Table 1**: Six hypersusceptible AD1-8u^-^ derivative strains (three uracil auxotrophic AD strains and three uracil prototrophic AD/pABC3 control strains) lacking seven major ABC transporters, including Pdr5, and two AD∆∆ strains overexpressing either the wild-type *CDR1* or the ‘catalytically inactive’ *CDR1*-*E1027Q* mutant, each with a C-terminal mGFPHis double tag that does not affect pump function.

**Table 1.** List of *Saccharomyces cerevisiae* strains used in these control experiments.

| **Strains** | **Genotype or description** | **Source** |
| --- | --- | --- |
| AD (AD1-8u^-^) | *MAT*a, *PDR1–3, ura3, his1, ∆yor1::hisG, ∆snq2::hisG, ∆pdr5::hisG, ∆pdr10::hisG, ∆pdr11::hisG, ∆ycf1::hisG, ∆pdr3::hisG, ∆pdr15::hisG* | (1) |
| AD∆ | AD1-8u^-^, *∆ura3* | (2) |
| AD∆∆ | AD1-8u^-^, *∆ura3*, *∆his1* | (3) |
| AD/pABC3 | AD1-8u^-^, *Δpdr5::pABC3* (empty vector control) | (2) |
| AD∆/pABC3 | AD∆, *Δpdr5::pABC3* (empty vector control) | (2) |
| AD∆∆/pABC3 | AD∆∆, *Δpdr5::pABC3* (empty vector control) | (4) |
| AD∆∆/CaCDR1A-GH | AD∆∆, *Δpdr5::CaCDR1A-GFP-HIS* | (5) |
| AD∆∆/CaCDR1A-E1027Q-GH | AD∆∆, *Δpdr5::CaCDR1A-E1027Q-GFP-HIS* | (5) |

To ensure the reliability and reproducibility of our crude PM preparation protocol, we evaluated:

(i) Whether 1 h glucose starvation on ice prior to cell harvest affects Cdr1 expression levels and/or ATPase activities; and (ii) Whether freeze-thawing and duration of crude PM storage at -20 ˚C affect the ATPase activities, and if so, by how much.

Glucose starvation on ice for 1 h is used routinely for the isolation of crude PMs which dramatically reduces the background Pma1 ATPase activity (6). For each strain, three independent PM preparations of logarithmic cells starved for 1 h on ice before cell harvest (i.e. starved) or harvested without glucose starvation (i.e. not starved) were obtained (in total 48 crude PM samples). These samples were then subjected to SDS-PAGE to quantify the Cdr1 expression levels, and their ATPase activities were measured as technical duplicates in the i) absence; or presence of ii) 40 μM oligomycin (OLI); or iii) 400 μM vanadate (VAN).

**SDS-PAGE analysis of crude plasma membrane (PM) samples – Cdr1 expression affects Pma1 expression levels.**

SDS-PAGE analysis of three biological replicates confirmed that 1 h glucose starvation on ice did not visibly affect the protein profiles of crude PMs, and the expression levels of the ‘catalytically inactive’ Cdr1-E1027Q mutant were comparable to wild-type Cdr1 overexpressing cells. However, overexpression of Cdr1, no matter whether catalytically active (wild-type Cdr1) or not (Cdr1-E1027Q), reduced the expression levels of the prominent PM proton pump, Pma1, by approximately half (**Fig. 1**).

There were no other visible differences between the various PM protein profiles, indicating the 1 h starvation at 4 ˚C does not affect the protein profile of crude PMs isolated with the optimized small-scale PM preparation protocol. These observations indicate that the exceptionally high expression levels of Cdr1 appear to compete with Pma1 for either the limited space available in the plasma membrane or for the ATP that is required for the pump activities of both enzymes. However, the fact that the catalytically inactive mutant affected the same reduction of the Pma1 expression levels as wild-type Cdr1 did suggests that both enzymes (i.e. Pma1 and Cdr1) indeed compete for the limited space available in the plasma membrane.


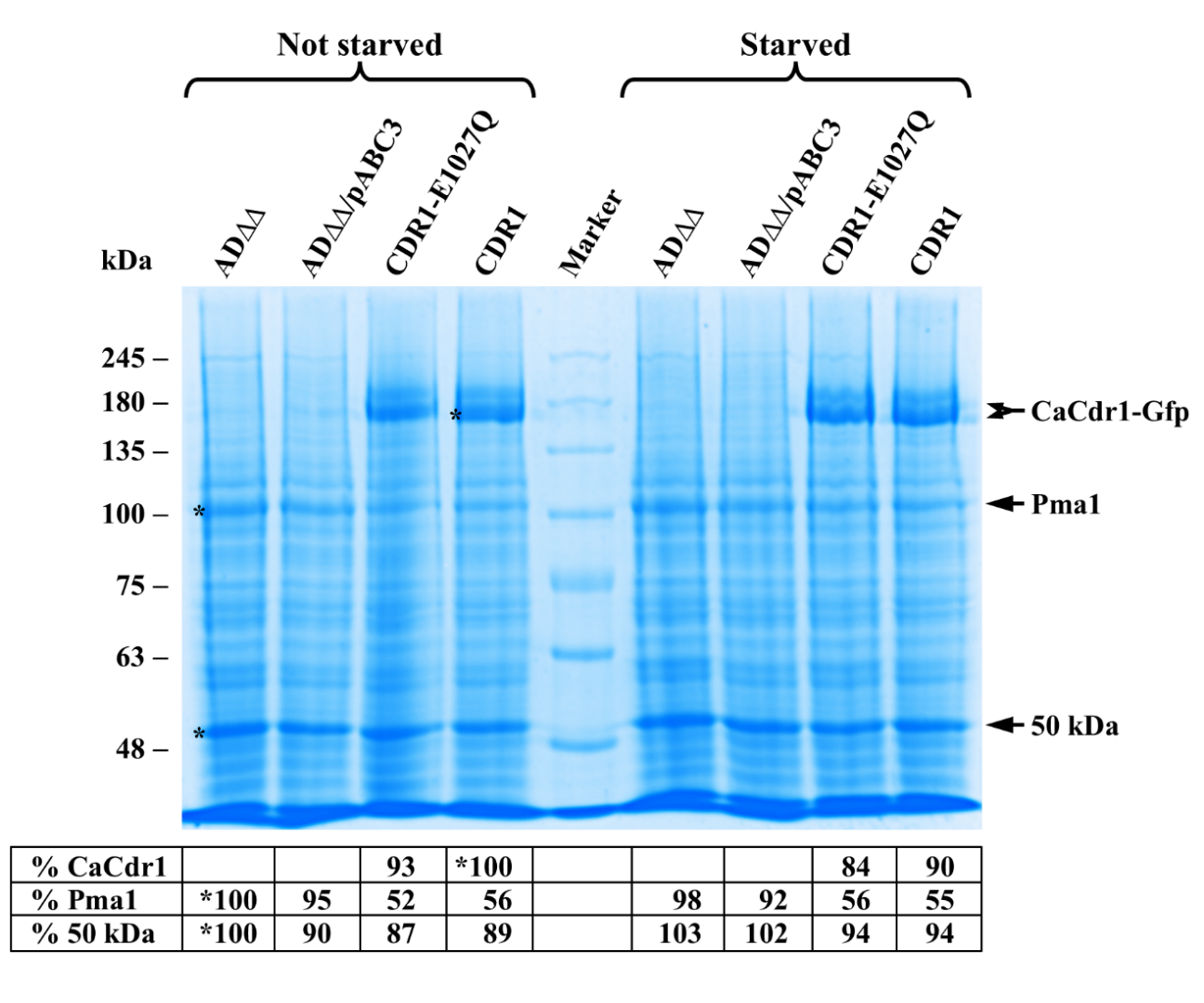


**Figure 1.** A representative SDS-PAGE of 30 μg crude PM proteins isolated from AD∆∆, AD∆∆/pABC3, AD∆∆/CaCDR1A-GH and AD∆∆/CaCDR1A-E1027Q-GH cells harvested immediately (not starved) or starved for 1 h on ice (starved) before cell harvest. Arrows indicate the ~200 kDa CaCdr1-Gfp double band (CaCdr1-Gfp runs as a double band because most of the C-terminal Gfp tag was not fully denatured even in the presence of 2% SDS and after 5 min denaturation at 50 ˚C), the ~110 kDa Pma1 band and the prominent ~50 kDa protein band that was used as a loading control. The amounts of protein were quantified with ImageJ (7). SDS-PAGE was undertaken three times with independently isolated PM protein samples and the average % expression levels of CaCdr1, ScPma1, and the ~50 kDa control protein relative to the indicated control bands (*) are listed underneath the Coomassie Blue stained image.

Similar observations have previously been made for the overexpression of several fungal PDR transporters, including Pdr5 and *C. albicans* Cdr2 (see **Fig. 4C** in reference (2)). It would seem that this competitive effect between PDR transporter and Pma1 expression levels is specific for Pma1; no other PM proteins were noticeably affected by the overexpression of Cdr1 (**Fig. 1**). As expected, tuning down the expression levels of Cdr1 and Pdr5 by placing well-defined GC-rich stem-loops adjacent to the AUG start codon of their mRNA transcripts had the opposite effect on the Pma1 expression levels (see **Figs. 1C** and **3A** in reference (8)). The results presented in **Fig. 3A** of reference (8) also clearly demonstrate that despite these exceptionally high Cdr1 expression levels, all Cdr1 molecules are fully functional and they are also properly localized to the PM (2). This was clearly demonstrated by the fact that the fluconazole (FLC) resistance levels (i.e. the MICs for FLC), a proxy for Cdr1 pump function, increased proportionally with increasing Cdr1 expression levels no matter how high the Cdr1 expression levels reached (8).

**Characterization of the ATPase activities of crude PMs of the eight AD test strains.**

The ATPase activities of freshly prepared crude PMs were compared with frozen PM aliquots stored at -20 °C for 4, 7, or 10 days, each PM aliquot was subjected to a single freeze-thaw cycle. For each strain and harvest condition, assays were performed as technical duplicates with one (fresh samples), two (7 days), or three (4 days and 10 days) independently prepared PM samples.

The following ATPase activities were determined for each PM aliquot: total, oligomycin-sensitive (OLI-S), oligomycin-insensitive (OLI-IS), vanadate-sensitive (VAN-S), vanadate-insensitive (VAN-IS), Cdr1-specific, and Pma1-specific. OLI-S and VAN-S ATPase activities were calculated by subtracting the ATPase activities remaining after treatment with 40 µM OLI and 400 µM VAN from the total ATPase activity, respectively. The Cdr1-specific ATPase activity was defined as the OLI-S ATPase activity minus the background OLI-S ATPase activity of the negative AD control strain(s). The Pma1-specific ATPase activity was defined as the VAN-S but OLI-IS ATPase activity (i.e., Pma1 = VAN-S minus OLI-S ATPase activity) (9).

No significant differences in ATPase activities were observed among the six AD strains under any of the test conditions (**Table 2**). The ATPase activities of crude PMs prepared under identical starvation and storage conditions were quite reproducible. However, there were noticeable differences between starved and unstarved cells and between freshly prepared and frozen PM aliquots of the same PM preparation (**Table 2**). The total, OLI-S, VAN-S, VAN-IS, Cdr1-specific, and Pma1-specific ATPase activities are listed in **Table 3** of the main manuscript.

**Table 2**. ATPase activities of fresh or frozen PM aliquots stored for 4, 7, or 10 days at -20 ˚C. Crude PMs were isolated from cells of six different AD1-8u^-^ control strains (three without and three with the *URA3* selection marker of the empty vector) that had either been starved for 1 h on ice, or immediately harvested before the crude PM preparation.

| **Strains** | | **Ura^-^** | | | | | | **Ura^+^** | | | | | | **ADs^b^** | |
| --- | --- | --- | --- | --- | --- | --- | --- | --- | --- | --- | --- | --- | --- | --- | --- |
|  |  | **AD** | | **ADΔ** | | **ADΔΔ** | | **AD/**  **pABC3** | | **ADΔ/**  **pABC3** | | **ADΔΔ/**  **pABC3** | |  |  |
| **Cells** | **PMs^a^** | **Total ATPase activity (nmol Pi/min/mg)** | | | | | | | | | | | | | |
| Not starved | Fresh | 242 | (-)^c^ | 234 | (-) | 252 | (-) | 222 | (-) | 245 | (-) | 265 | (-) | 243 | (15) |
|  | 4 days | 151 | (17) | 145 | (10) | 145 | (21) | 138 | (16) | 151 | (17) | 162 | (16) | 149 | (16) |
|  | 7 days | 116 | (9) | 118 | (3) | 105 | (11) | 114 | (4) | 117 | (1) | 126 | (5) | 116 | (8) |
| Starved | Fresh | 106 | (-) | 101 | (-) | 98 | (-) | 99 | (-) | 116 | (-) | 115 | (-) | 106 | (8) |
|  | 4 days | 80 | (19) | 74 | (13) | 75 | (14) | 76 | (20) | 84 | (16) | 84 | (14) | 79 | (14) |
|  | 7 days | 70 | (6) | 73 | (4) | 79 | (7) | 91 | (3) | 82 | (1) | 76 | (12) | 78 | (9) |
|  | 10 days | nd^d^ |  | nd |  | 70 | (13) | nd |  | nd |  | 61 | (12) | 66 | (12) |
| **Cells** | **PMs^a^** | **OLI-S ATPase activity (nmol Pi/min/mg)^e^** | | | | | | | | | | | | | |
| Not starved | Fresh | 31 | (-) | 47 | (-) | 55 | (-) | 39 | (-) | 46 | (-) | 40 | (-) | 43 | (8) |
|  | 4 days | 14 | (13) | 21 | (7) | 21 | (20) | 15 | (9) | 22 | (13) | 19 | (11) | 18 | (11) |
|  | 7 days | -2 | (9) | 5 | (1) | 0 | (1) | 9 | (7) | 3 | (1) | 1 | (3) | 3 | (5) |
| Starved | Fresh | 25 | (-) | 31 | (-) | 28 | (-) | 30 | (-) | 29 | (-) | 29 | (-) | 29 | (2) |
|  | 4 days | 21 | (9) | 21 | (5) | 21 | (7) | 19 | (15) | 16 | (6) | 17 | (6) | 19 | (8) |
|  | 7 days | 13 | (8) | 16 | (12) | 22 | (1) | 25 | (6) | 15 | (7) | 7 | (2) | 16 | (8) |
|  | 10 days | nd |  | nd |  | 21 | (5) | nd |  | nd |  | 2 | (4) | 11 | (11) |
| **Cells** | **PMs^a^** | **VAN-S ATPase activity (nmol Pi/min/mg)^f^** | | | | | | | | | | | | | |
| Not starved | Fresh | 177 | (-) | 165 | (-) | 176 | (-) | 150 | (-) | 172 | (-) | 188 | (-) | 172 | (13) |
|  | 4 days | 88 | (10) | 80 | (1) | 79 | (10) | 75 | (7) | 84 | (6) | 95 | (10) | 83 | (10) |
|  | 7 days | 66 | (6) | 51 | (5) | 51 | (4) | 53 | (10) | 59 | (2) | 68 | (8) | 58 | (9) |
| Starved | Fresh | 49 | (-) | 44 | (-) | 41 | (-) | 40 | (-) | 62 | (-) | 57 | (-) | 49 | (9) |
|  | 4 days | 17 | (2) | 16 | (2) | 16 | (2) | 18 | (4) | 27 | (1) | 23 | (2) | 19 | (5) |
|  | 7 days | 16 | (2) | 14 | (3) | 12 | (1) | 16 | (1) | 24 | (4) | 20 | (3) | 17 | (5) |
|  | 10 days | nd |  | nd |  | 10 | (1) | nd |  | nd |  | 21 | (4) | 15 | (6) |
| **Cells** | **PMs^a^** | **VAN-IS ATPase activity (nmol Pi/min/mg)** | | | | | | | | | | | | | |
| Not starved | Fresh | 65 | (-) | 68 | (-) | 75 | (-) | 72 | (-) | 73 | (-) | 77 | (-) | 72 | (4) |
|  | 4 days | 63 | (13) | 66 | (9) | 66 | (15) | 63 | (11) | 68 | (13) | 67 | (10) | 65 | (10) |
|  | 7 days | 50 | (3) | 67 | (2) | 54 | (8) | 60 | (7) | 58 | (1) | 58 | (4) | 58 | (6) |
| Starved | Fresh | 57 | (-) | 57 | (-) | 57 | (-) | 58 | (-) | 54 | (-) | 58 | (-) | 57 | (2) |
|  | 4 days | 63 | (21) | 58 | (15) | 59 | (17) | 58 | (16) | 57 | (17) | 61 | (16) | 59 | (14) |
|  | 7 days | 54 | (4) | 59 | (2) | 68 | (8) | 75 | (3) | 58 | (4) | 55 | (9) | 61 | (9) |
|  | 10 days | nd |  | nd |  | 60 | (13) | nd |  | nd |  | 41 | (8) | 50 | (14) |
| **Cells** | **PMs^a^** | **Pma1 ATPase activity (nmol Pi/min/mg)^h^** | | | | | | | | | | | | | |
| Not starved | Fresh | 146 | (-) | 118 | (-) | 122 | (-) | 112 | (-) | 127 | (-) | 148 | (-) | 129 | (15) |
|  | 4 days | 74 | (15) | 59 | (7) | 58 | (21) | 60 | (11) | 62 | (14) | 77 | (17) | 65 | (14) |
|  | 7 days | 68 | (15) | 46 | (4) | 51 | (3) | 45 | (18) | 56 | (2) | 67 | (11) | 55 | (12) |
| Starved | Fresh | 24 | (-) | 13 | (-) | 13 | (-) | 10 | (-) | 33 | (-) | 28 | (-) | 20 | (10) |
|  | 4 days | -5 | (11) | -5 | (6) | -5 | (9) | -1 | (11) | 12 | (6) | 6 | (8) | 0 | (10) |
|  | 7 days | 3 | (10) | -2 | (9) | -11 | (1) | -9 | (6) | 9 | (11) | 13 | (1) | 1 | (11) |
|  | 10 days | nd |  | nd |  | -11 | (5) | nd |  | nd |  | 18 | (2) | 4 | (16) |

^a^ Freshly prepared PMs and frozen PM aliquots stored at -20 °C for 4, 7, or 10 days.

^b^ These are the mean values and in brackets the +/- SDs of all six AD strains combined.

^c^ The data are the means (+/- the SDs). The (-) sign means that there are no SDs because only one data point exists.

^d^ nd = not determined.

^e^ The OLI-S ATPase activities were measured and calculated in the presence of 40 µM OLI.

^f^ The VAN-S ATPase activities were measured and calculated in the presence of 400 µM VAN.

^h^ Pma1 ATPase activity = the VAN-S ATPase activity minus the OLI-S ATPase activity.

**The ATPase activities of the six AD test strains are indistinguishable from each other.**

The results presented in **Table 2** and **Figs. 2-4** clearly demonstrate that there are no noticeable differences between the ATPase activities of any of the six AD control strains. This means that the presence of the *URA3* gene and the uracil prototroph phenotype of the three AD/pABC3 test strains does not affect the background ATPase activities of any of the six AD test strains. Thus, any of the six AD strains could be used as a negative control to determine the Cdr1-specific ATPase activity of strains overexpressing Cdr1, as long as the overexpression of Cdr1 itself does not affect any of these background ATPase activities.


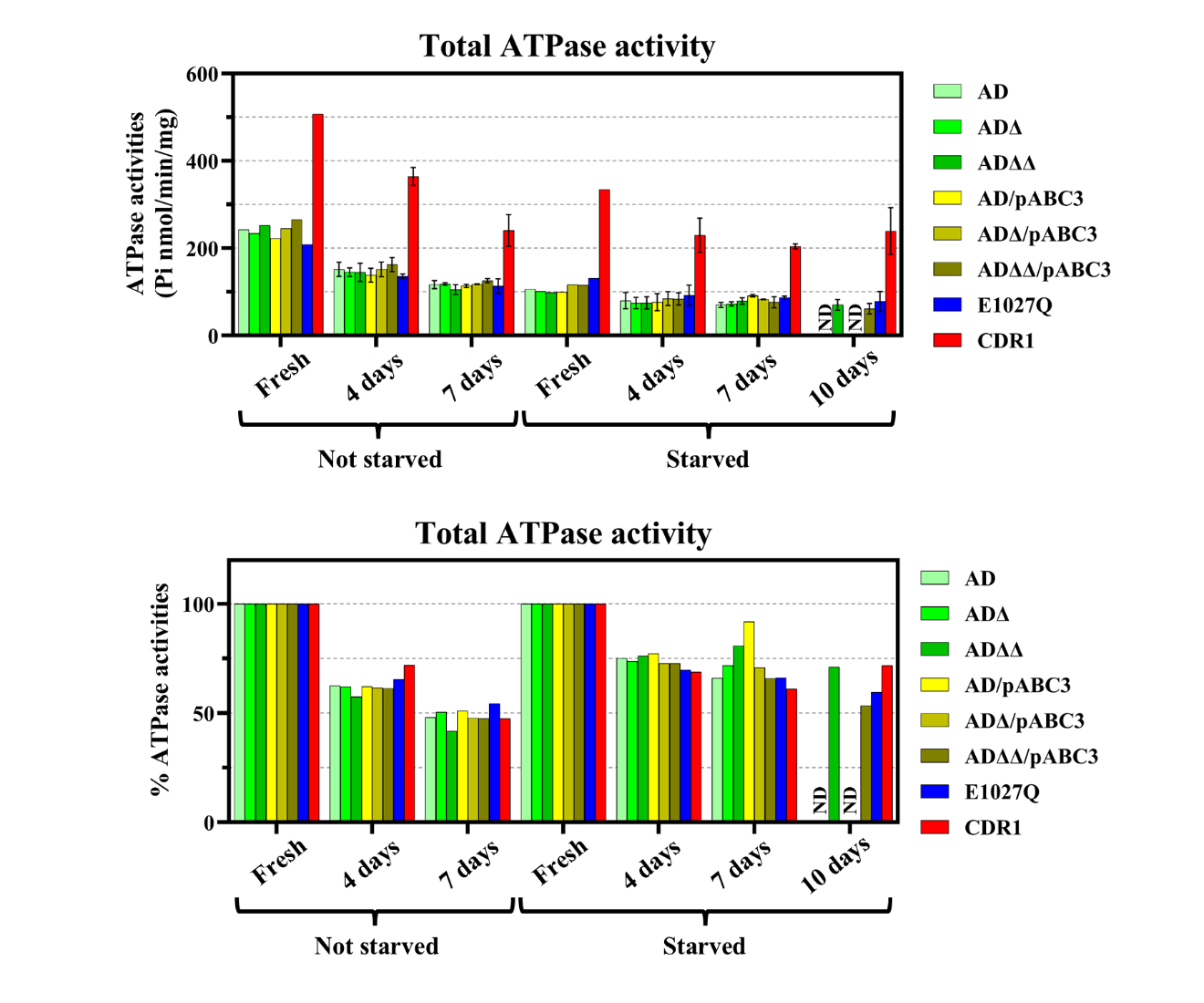


**Figure 2.** Influence of glucose starvation, the quality (freezing), and the length of frozen storage on the total ATPase activities of crude PMs isolated from the six AD control strains, and from AD∆∆ strains overexpressing either wild-type Cdr1 or Cdr1-E1027Q. The percentage of ATPase activities of the indicated strains normalized against the ATPase activities of fresh PMs (100%) is shown in the lower panel. The data represent the means ± SD of technical duplicates of one (Fresh), two (7 days), or three (4 days and 10 days) independently isolated PM samples. The ATPase activities of the crude PM samples of only four of the eight strains (i.e. AD∆∆, AD∆∆/pABC3, CDR1, and E1027Q) were determined after storage for 10 days (ND = not determined). The ATPase activity of one 70 µL PM aliquot was measured immediately (Fresh), while the remaining 70 µL aliquots were stored frozen at -20 °C for 4, 7, or 10 days before determining their ATPase activities.


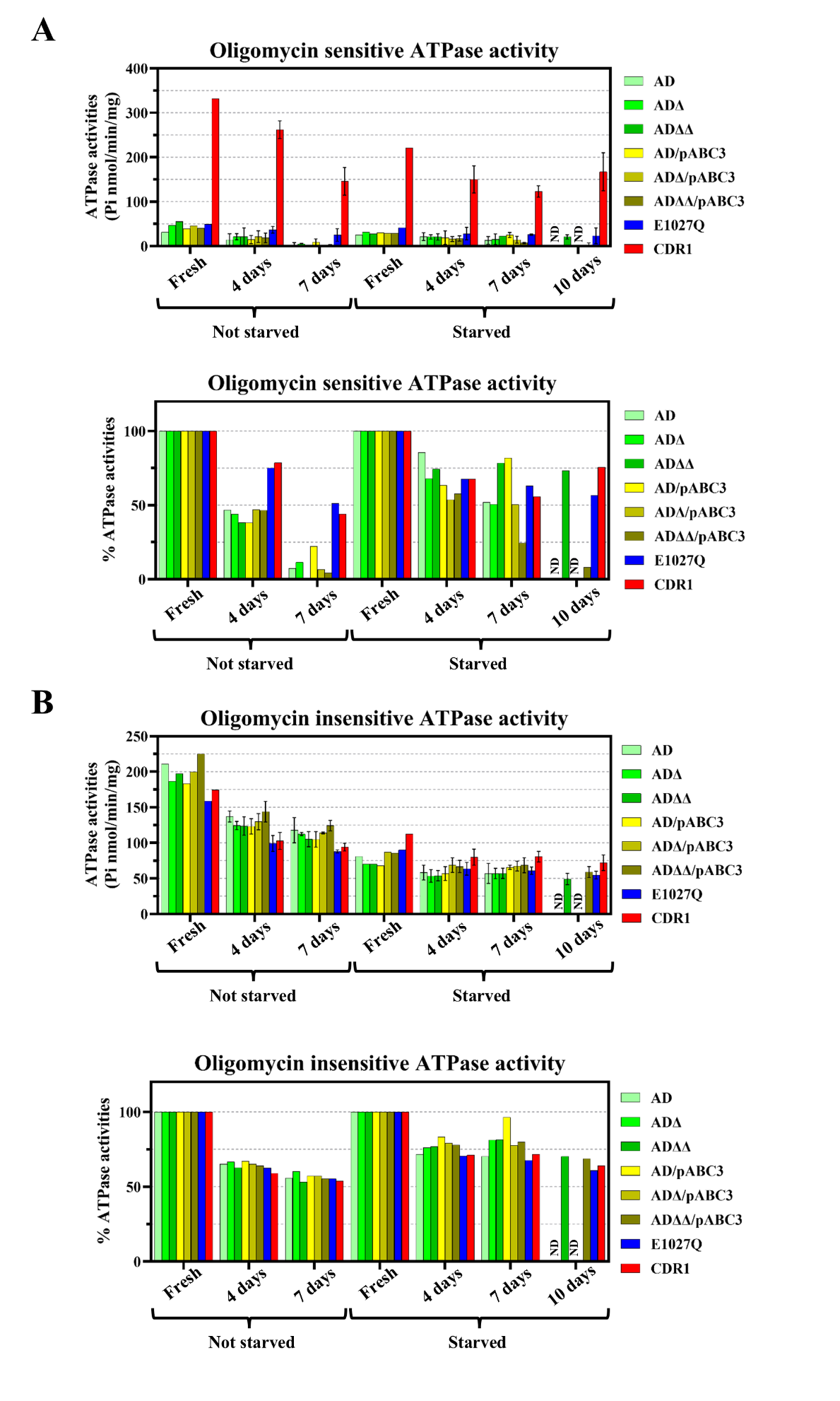


**Figure 3.** Influence of glucose starvation, the quality (freezing), and the length of frozen storage on the OLI-S ATPase activities of crude PMs isolated from the indicated strains. The percentages of ATPase activities normalized against the ATPase activities of fresh PMs (100%) are shown in the lower panel. The data represent the means ± SD of technical duplicates of one (Fresh), two (7 days), or three (4 days and 10 days) independently isolated PM samples. ND = not determined.


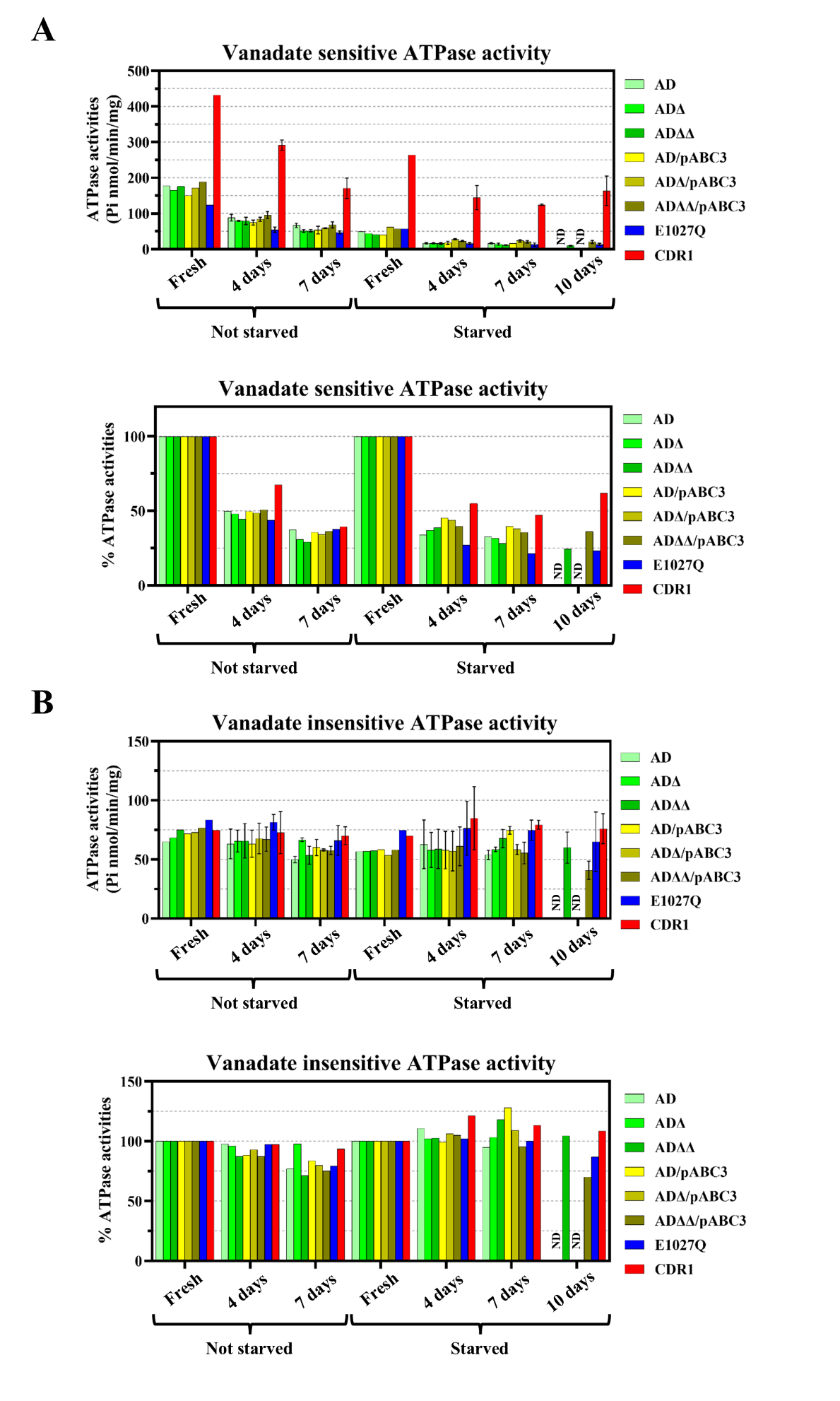


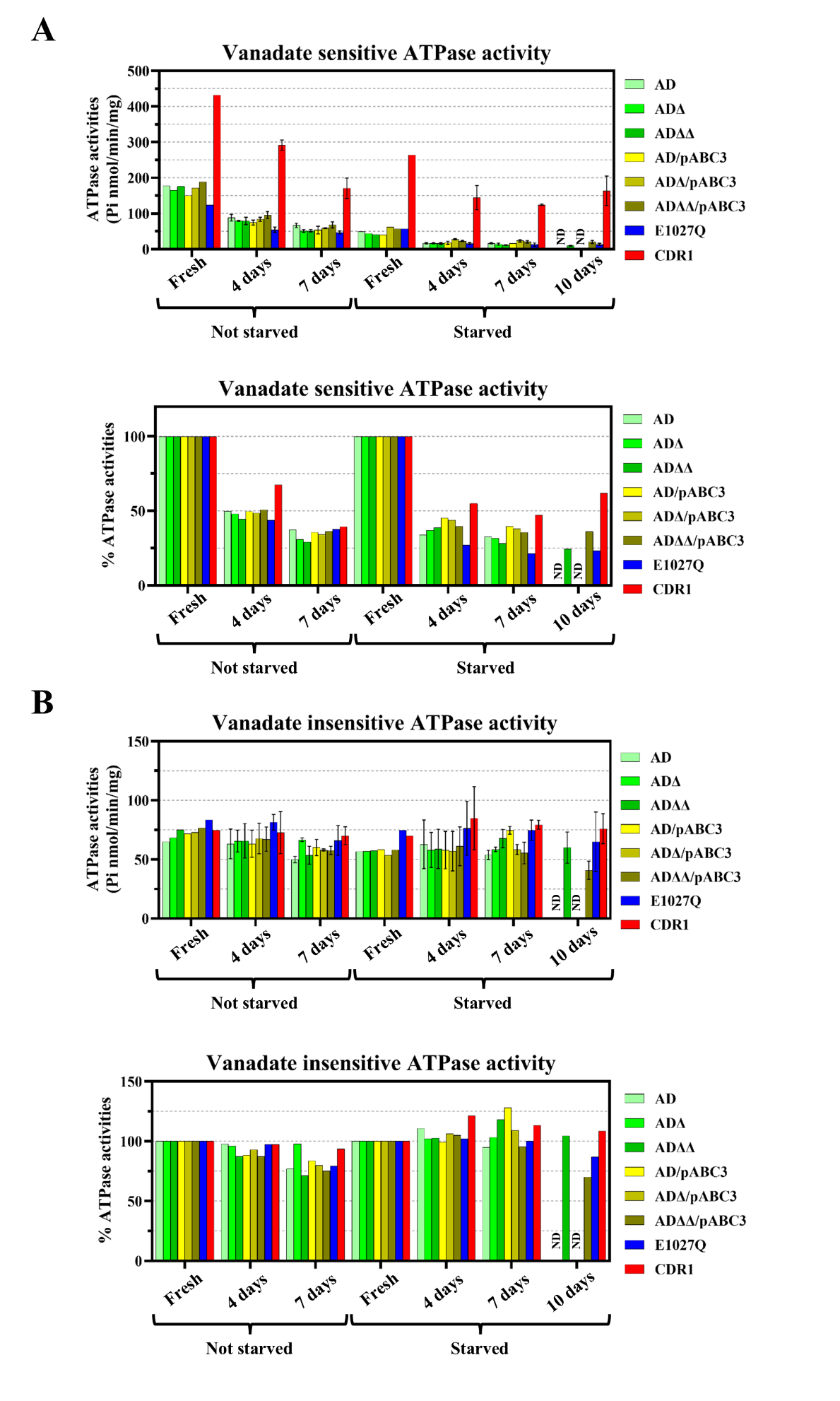


**Figure 4.** Influence of glucose starvation, the quality (freezing), and the length of frozen storage on the VAN-S **(A)** and VAN-IS **(B)** ATPase activities of crude PMs isolated from the indicated strains. The percentages of ATPase activities normalized against those of fresh PMs (100%) are shown in the two lower panels. The data represent the means ± SD of technical duplicates of one (Fresh), two (7 days), or three (4 days and 10 days) independently isolated PM samples. ND = not determined.

**Pma1 and VAN-IS background ATPase activities.**

The total, OLI-S, and VAN-S PM ATPase activities of all eight test strains were significantly lower (~50 %) in PMs that were obtained from glucose-starved cells (**Table 2** and **Figs. 2-4**). However, the VAN-IS PM ATPase activities of all eight test strains were indistinguishable from each other and largely unaffected by starvation, freezing, or storage duration (**Fig. 4B**). This led to several important observations: i) As expected from the ~45 % reduced Pma1 expression levels caused by the overexpression of Cdr1 (**Fig. 1**), the Pma1-specific ATPase activities (112-148 nmol Pi/min/mg; **Table 2**) of fresh PMs from any of the six non-starved AD strains were higher (by 11-49 %) than those (75 and 100 nmol Pi/min/mg) of the Cdr1 overexpressing strains, (Cdr1 active or inactive) (**Fig. 5**); and ii) Freeze-thaw treatment and storage significantly reduced those Pma1 ATPase activities to ~25-50 % (**Fig. 5**).

Most importantly, however: i) Glucose starvation reduced the Pma1-specific ATPase activities of fresh PMs from all of the eight test strains to very low levels (10-42 nmol Pi/min/mg) after just one additional freeze-thaw cycle (**Fig. 5**); and ii) The VAN-IS background ATPase activities were indistinguishable from each other in all eight test strains under all tested growth and storage conditions (**Fig. 4B**).


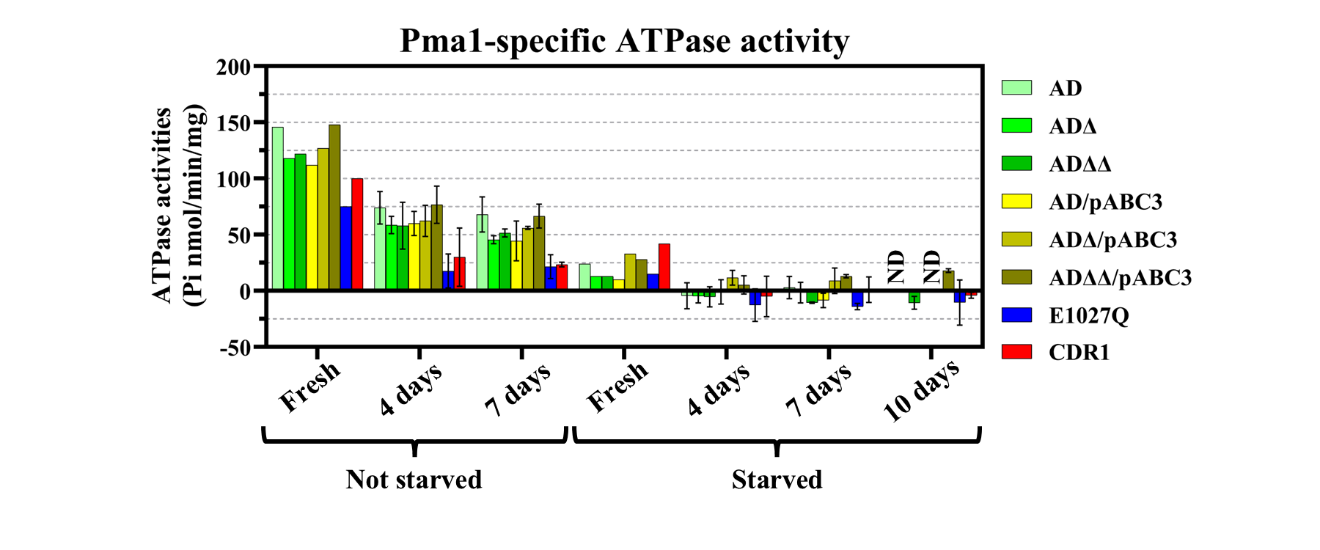


**Figure 5.** Influence of glucose starvation, the quality (freezing), and the length of frozen storage on the Pma1-specific ATPase activities of crude PMs isolated from the indicated strains. The data represent the means ± SD of technical duplicates of one (Fresh), two (7 days), or three (4 days and 10 days) independently isolated PM samples. ND = not determined.

**Cdr1-E1027Q has residual OLI-S ATPase activity that is VAN-IS but overexpression of Cdr1-E1027Q does not affect the OLI-S background ATPase activity of the AD strains.**

The OLI-S ATPase activities of the six AD negative control strains were rather low, even in freshly prepared PM samples of cells that were not starved (31-55 nmol Pi/min/mg) which were slightly higher than the OLI-S PM ATPase activities (25-31 nmol Pi/min/mg) of starved cells (**Table 2** and **Fig. 3**). The OLI-S PM ATPase activities of the cells that were not starved decreased to undetectable levels after 7 days storage while the OLI-S PM ATPase activities of the starved cells decreased to almost undetectable levels (<20 nmol Pi/min/mg; **Table 2** and **Fig. 3**). Interestingly, the OLI-S background ATPase activity of freshly prepared PMs of the catalytically inactive Cdr1-E1027Q variant, starved or unstarved, were indistinguishable from those of the AD control strains (**Fig. 3**).

This indicates that the overexpression of Cdr1 does not affect the OLI-S background ATPase activity of any of the AD strains. However, the OLI-S ATPase activities of Cdr1-E1027Q did not decrease to undetectable levels, starved or unstarved, and no matter how long their PMs were stored, remaining consistently at ~10-20 nmol Pi/min/mg. This is possibly because Cdr1-E1027Q has some, although very low, residual OLI-S ATPase activity that is apparently less susceptible to freezing and long-term storage than the OLI-S background ATPase activity of the AD strains (**Fig. 3**). Interestingly, it appears that this residual OLI-S ATPase activity of Cdr1-E1027Q is VAN-IS. This is possibly the reason why the ‘Pma1’ PM ATPase activities of the Cdr1-E1027Q strain reached negative values (-10 to -15 nmol Pi/min/mg) in crude PM samples that were stored for more than 4 days (**Fig. 5**). Golnoush Madani reported a similarly low but above background ATPase activity for the same Cdr1-E1027Q variant in her PhD thesis, confirming our findings.

**Cdr1-specific ATPase activities calculated in three different ways give very similar results.**

In this manuscript, the Cdr1-specific ATPase activities were determined by subtracting the total ATPase activity of the negative AD/pABC3 control strain from the total ATPase activity of the AD strain overexpressing wild-type Cdr1 (AD/CDR1B) or Cdr1 mutant variants.

This choice was made because some Cdr1 mutants were OLI and/or VAN resistant. As shown in **Fig. 6**, the Cdr1-specific ATPase activities of wild-type Cdr1 using either AD strains or the Cdr1-E1027Q mutant as background were nearly identical regardless of the calculation method (**Fig. 6**). Overexpression of Cdr1 did not alter any of the background ATPase activities of frozen crude PM aliquots isolated from AD cells starved for 1 h on ice prior to cell harvest.


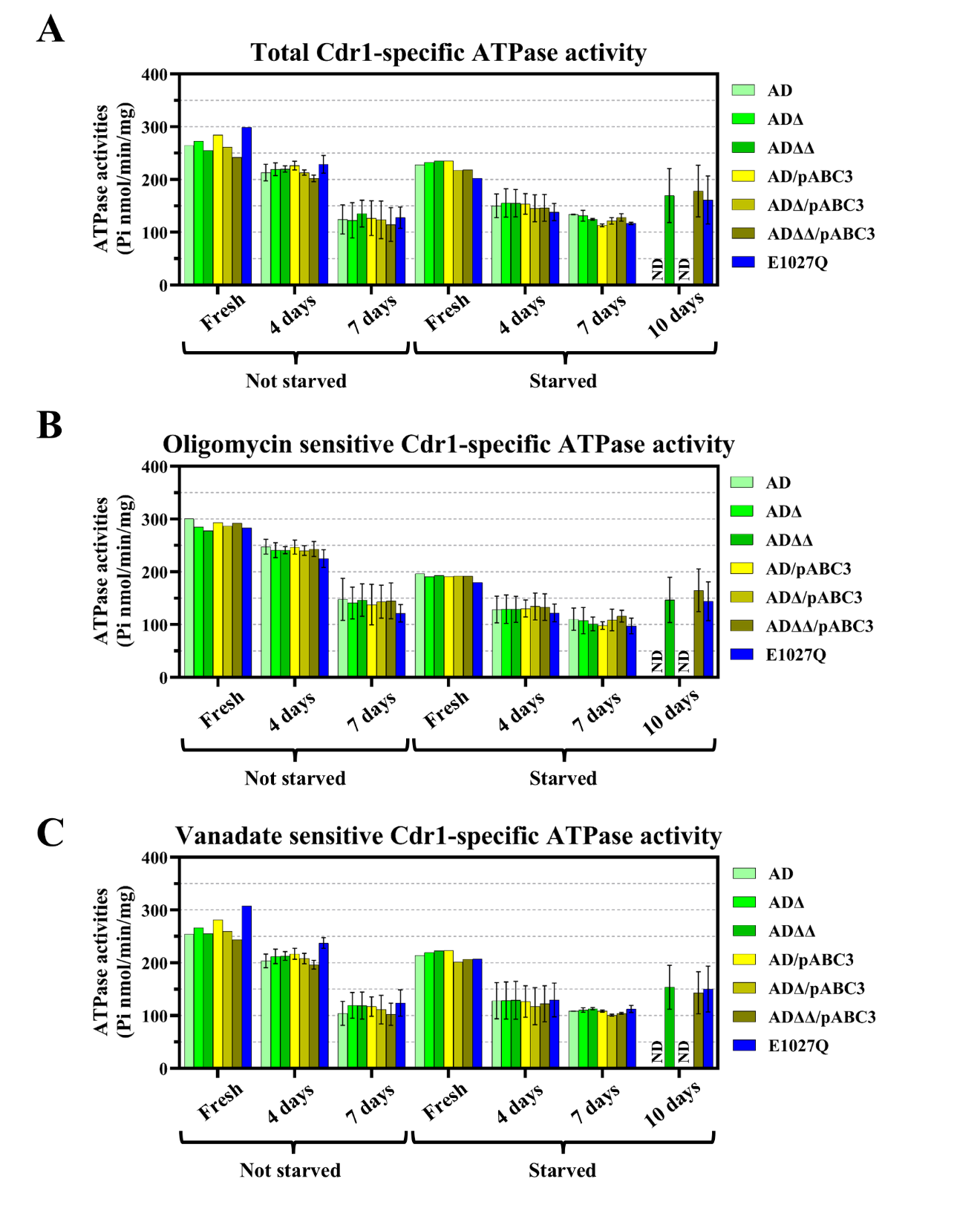


**Figure 6.** Cdr1-specific ATPase activities calculated in three different ways: **(A)** Total; **(B)** OLI-S; and **(C)** VAN-S ATPase activities of wild-type Cdr1 minus the total, the OLI-S, and the VAN-S ATPase activities of the six AD control strains or E1027Q mutant, respectively. The data were obtained from the raw data presented in **Figures 2, 3 and 4.** They are the means ± SD of technical duplicates of one to three independently isolated PM samples. ND = not determined.

**Conclusion.** The Cdr1-specific ATPase activity can be accurately determined by simply subtracting the background ATPase activity of any AD host strain, provided that all PMs are prepared from cells starved for 1 h on ice and they are stored frozen for an identical period of time, but for no longer than 1 or 2 weeks. Furthermore, our investigations also highlight the fact that the Cdr1-specific ATPase activity of crude PMs is quite sensitive to physical changes of the plasma membrane lipid bilayer caused by freezing and long-term storage. We also conclude that the PM background ATPase activity of any negative AD control strain is a more suitable control than the ‘catalytically’ inactive Cdr1-E1027Q mutant which itself has residual ATPase activity.

**References**

1. Decottignies A, Grant AM, Nichols JW, de Wet H, McIntosh DB, Goffeau A. 1998. ATPase and multidrug transport activities of the overexpressed yeast ABC protein Yor1p. J Biol Chem 273:12612-22.

2. Lamping E, Monk BC, Niimi K, Holmes AR, Tsao S, Tanabe K, Niimi M, Uehara Y, Cannon RD. 2007. Characterization of three classes of membrane proteins involved in fungal azole resistance by functional hyperexpression in *Saccharomyces cerevisiae*. Eukaryot Cell 6:1150-65.

3. Sagatova AA, Keniya MV, Wilson RK, Monk BC, Tyndall JD. 2015. Structural insights into binding of the antifungal drug fluconazole to *Saccharomyces cerevisiae* lanosterol 14alpha-demethylase. Antimicrob Agents Chemother 59:4982-9.

4. Madani G, Lamping E, Cannon RD. 2021. Engineering a cysteine-deficient functional *Candida albicans* Cdr1 molecule reveals a conserved region at the cytosolic apex of ABCG transporters important for correct folding and trafficking of Cdr1. mSphere 6:e01318-20

5. Madani G. 2020. Biochemical and structural analysis of *Candida albicans* multidrug efflux pump Cdr1. PhD thesis. University of Otago, Dunedin New Zealand.

6. Serrano R. 1983. In vivo glucose activation of the yeast plasma membrane ATPase. FEBS Lett 156:11-4.

7. Schneider CA, Rasband WS, Eliceiri KW. 2012. NIH Image to ImageJ: 25 years of image analysis. Nat Methods 9:671-5.

8. Lamping E, Niimi M, Cannon RD. 2013. Small, synthetic, GC-rich mRNA stem-loop modules 5' proximal to the AUG start-codon predictably tune gene expression in yeast. Microb Cell Fact 12:74.

9. Keniya MV, Cannon RD, Nguyen A, Tyndall JD, Monk BC. 2013. Heterologous expression of *Candida albicans* Pma1p in *Saccharomyces cerevisiae*. FEMS Yeast Res 13:302-11.
