# Supplementary material for: G521 is the gatekeeper and a key transmembrane domain contact residue of Candida albicans Cdr1
Source: mBio. 2026 Feb 26;17(4):e03746-25. doi: 10.1128/mbio.03746-25 (PMC13059742; doi:10.1128/mbio.03746-25)
Supplement: Supplemental material — Supplemental tables and figures. [file mbio.03746-25-s0002.docx]

**Supplementary Table S1.** MICs^a^ of 11 antifungals for *S. cerevisiae* AD test strains.

| **Strain^b^** | **CER^c^** | **CHX** | **FLC** | **VRC** | **MCZ** | **R6G** | **KTC** | **MON** | **POS** | **ITC** | **NIG** |
| --- | --- | --- | --- | --- | --- | --- | --- | --- | --- | --- | --- |
|  | **223^d^** | **281** | **306** | **349** | **416** | **479** | **531** | **671** | **701** | **706** | **725** |
| AD/pABC3 | 0.25 | 0.016 | 1 | 0.016 | 0.008 | 1 | 0.004 | 0.5 | 0.063 | 0.031 | 0.25 |
| AD/CDR1B | 4 | 2 | 512 | 16 | 8 | 64 | 8 | 256 | >32 | >32 | 64 |
| G521R | 4 | 1 | 128 | 1 | 2 | 4 | 0.125 | 1 | 0.063 | 0.25 | 0.25 |
| *R521L | 16 | 8 | 1024 | 32 | 16 | 16 | 8 | 256 | >32 | >32 | 16 |
| *R521P | 4 | 1 | 512 | 8 | 4 | 32 | 2 | 256 | >32 | >32 | 8 |
| *R521C | 16 | 4 | 1024 | 16 | 8 | 64 | 4 | 256 | >32 | >32 | 64 |
| *R521S | 16 | 4 | 1024 | 32 | 8 | 128 | 4 | 256 | >32 | >32 | 16 |
| G521D | 16 | 4 | 1024 | 4 | 8 | 4 | 2 | 16 | 0.5 | 1 | 4 |
| G521F | 16 | 1 | 128 | 0.5 | 0.5 | 1 | 0.5 | 8 | 0.25 | 1 | 2 |
| *F521/D511V | 4 | 2 | 512 | 4 | 2 | 4 | 4 | 64 | 2 | >32 | 16 |
| *F521/A549V | 4 | 2 | 256 | 0.5 | 2 | 16 | 2 | 8 | 2 | >32 | 2 |
| *F521/T658P | 8 | 2 | 256 | 2 | 4 | 16 | 2 | 32 | 0.5 | 8 | 8 |
| **F521/G672R | 0.5 | 0.125 | 16 | 0.125 | 1 | 32 | 0.25 | 2 | 0.125 | 1 | 0.5 |
| G521H | 4 | 2 | 256 | 1 | 1 | 2 | 1 | 4 | 0.25 | 1 | 0.5 |
| **H521/A548G | 8 | 2 | 256 | 1 | 2 | 64 | 2 | 4 | 0.25 | 1 | 1 |
| **H521/C712S | 2 | 1 | 128 | 0.5 | 1 | 32 | 1 | 1 | 0.5 | 2 | 0.5 |
| *H521/L782F | 4 | 1 | 512 | 4 | 8 | 4 | 4 | 4 | 0.5 | >32 | 1 |
| *H521/L1225I | 4 | 1 | 256 | 2 | 1 | 32 | 4 | 8 | 1 | >32 | 2 |
| G521W | 4 | 1 | 256 | 2 | 2 | 1 | 2 | 32 | 1 | 4 | 4 |
| *W521C | 16 | 4 | 1024 | 16 | 8 | 32 | 4 | 128 | 16 | >32 | 128 |
| *W521S | 16 | 4 | 1024 | 32 | 16 | 32 | 4 | 256 | 16 | >32 | 8 |
| **W521/R546G | 8 | 4 | 256 | 4 | 8 | 16 | 4 | 16 | >32 | >32 | 4 |
| *W521/A549V | 1 | 0.25 | 32 | 0.5 | 1 | 32 | 1 | 16 | 2 | >32 | 4 |
| *W521/V649F | 2 | 1 | 256 | 4 | 2 | 1 | 2 | 64 | 1 | 8 | 2 |
| G521Y | 8 | 1 | 128 | 0.25 | 1 | 1 | 0.5 | 4 | 0.25 | 1 | 0.25 |
| **Y521/G672A | 2 | 1 | 128 | 0.25 | 4 | 16 | 0.5 | 1 | 1 | 0.5 | 0.25 |

^a^ The units are mg/L.

^b^ Cdr1B “suppressor” mutants marked with one (*) or two (**) asterisks were selected on plates containing >2x MIC of ITC or R6G, respectively.

^c^ CER: Cerulenin, CHX: Cycloheximide, FLC: Fluconazole, VRC: Voriconazole, MCZ: Miconazole, R6G: Rhodamine 6G, KTC: Ketoconazole, MON: Monensin, POS: Posaconazole, ITC: Itraconazole, NIG: Nigericin.

^d^ The molecular weights (Da) of substrates are shown below the abbreviation.

**Supplementary Table S2.** Evaluation of statistically significant differences between the kinetic properties and the IC_50_ values of the ATPase activities of wild-type Cdr1 and the indicated Cdr1 variants presented in **Table 4**.

| **Strains** | **Cdr1 ATPase kinetic properties** | | | | | **Cdr1 ATPase IC_50_ values** | | | | | |
| --- | --- | --- | --- | --- | --- | --- | --- | --- | --- | --- | --- |
|  | ***V_max_*** | | ***K_m_*** | | | **Beauvericin** | | **Oligomycin** | | **Vanadate** | |
|  | **versus**  **WT** | **versus**  **parent** | **versus**  **WT** | | **versus**  **parent** | **versus**  **WT** | **versus**  **parent** | **versus**  **WT** | **versus parent** | **versus**  **WT** | **versus parent** |
| AD/CDR1B | 980 nmol/min/mg Cdr1^a^ | | 0.46 * 10^-3^ M | | | 0.3 μM | | 0.6 μM | | 1.2 μM | |
| G521D | ns | -- | ns | -- | | ↑*** | -- | ↑*** | -- | ↑*** | -- |
| G521W | ↑***^b^ | -- | ↑** | -- | | ↑*** | -- | ns | -- | ↑*** | -- |
| G521H | ↑*** | -- | ns | -- | | ↑*** | -- | ↑*** | -- | ↑*** | -- |
| H521/C712S | ↓* | ↓*** | ns | ns | | ↑*** | ns | ↑*** | ns | ↑*** | ns |
| H521/L1225I | ↑*** | ns | ns | ns | | ↑*** | ns | ↑*** | ↑** | ↑*** | ↓*** |
| G521F | ↑*** | -- | ↑*** | -- | | ↑*** | -- | ↑*** | -- | ↑*** | -- |
| F521/D511V | ↓*** | ↓*** | ns | ↓*** | | ↑*** | ↓** | ↑*** | ↓*** | ↑* | ↓*** |
| F521/A549V | ↑*** | ns | ns | ns | | ↑*** | ns | ↑*** | ↑*** | ↑*** | ↓*** |
| F521/T658P | ↑*** | ↓*** | ns | ↓*** | | ↑*** | ↓* | ↑*** | ↓*** | ns | ↓*** |
| F521/G672R | ↑* | ↓*** | ↓** | ↓*** | | ↑*** | ns | ↑*** | ↑*** | ↓** | ↓*** |

^a^ Values are calculated from three independent experiments and accounted for the different Cdr1 expression levels, based on the assumption that Cdr1 accounted for 15 % of the total crude PM protein in the wild-type strain (determined from SDS-PAGE analysis).

^b^ Arrows indicate significantly increased or decreased values compared to wild-type (WT) Cdr1 or the parental Cdr1 mutants G521F and G521H. Extra sum-of-squares F test was performed to evaluate whether the differences in parameters between the indicated strain and wild-type (WT) Cdr1 or the parental Cdr1 mutants G521F and G521H presented in **Table 4** are statistically significant (**p*<0.05, ***p*<0.01, ****p*<0.001, ns = not significant, -- = no data).

**Supplementary Table S3.** Evaluation of statistically significant differences between the whole-cell R6G transport parameters and the ATPase activities of wild-type Cdr1 and the indicated Cdr1 variants.

| **Strains** | **Whole-cell R6G transport assay parameters^a^** | | | | | | **ATPase activity** | |
| --- | --- | --- | --- | --- | --- | --- | --- | --- |
|  | **EQ** | | **MAX** | | **t_1/2_** | |  |  |
|  | **versus**  **WT** | **versus parent** | **versus**  **WT** | **versus parent** | **versus**  **WT** | **versus parent** | **versus**  **WT** | **versus parent** |
| AD/CDR1B | 101 % RFU | | 11 %∆RFU/min | | 5.5 min | | 940 nmol/min/mg Cdr1 | |
| G521R | ↓***^b^ | -- | ↓*** | -- | ↑*** | -- | ns | -- |
| R521L | ↓*** | ↑*** | ↓*** | ↑** | ↑** | ns | ↑** | ↑* |
| R521P | ns | ↑*** | ↓*** | ↑*** | ↑*** | ↓*** | ns | ↓* |
| R521C | ns | ↑*** | ↓*** | ↑*** | ↑* | ↓** | ↑* | ns |
| R521S | ↑*** | ↑*** | ns | ↑*** | ↑** | ↓* | ↑* | ns |
| G521D | ↓*** | -- | ↓*** | -- | ↑*** | -- | ns | -- |
| G521F | ↓*** | -- | ↓*** | -- | ns | -- | ↑*** | -- |
| F521/D511V | ↓*** | ↑*** | ↓*** | ↑*** | ↑*** | ns | ↓** | ↓*** |
| F521/A549V | ↓*** | ↑*** | ↓*** | ↑*** | ↑*** | ns | ↑*** | ns |
| F521/T658P | ↓*** | ↑*** | ↓*** | ↑*** | ↑*** | ns | ↑*** | ↓*** |
| F521/G672R | ↑*** | ↑*** | ns | ↑*** | ↑*** | ns | ↑* | ↓*** |
| G521H | ↓*** | -- | ↓*** | -- | ↑*** | -- | ↑*** | -- |
| H521/A548G | ↑** | ↑*** | ns | ↑*** | ↑* | ↓*** | ns | ↓* |
| H521/C712S | ↑*** | ↑*** | ↑*** | ↑*** | ns | ↓*** | ns | ↓*** |
| H521/L782F | ↑*** | ↑*** | ↓*** | ↑*** | ↑*** | ns | ns | ns |
| H521/L1225I | ↓* | ns | ↓*** | ↑*** | ↑** | ↓*** | ↑*** | ns |
| G521W | ↓*** | -- | ↓*** | -- | ↑*** | -- | ↑*** | -- |
| W521/R546G | ↓** | ↑*** | ↓*** | ↑*** | ↑*** | ↓*** | ns | ns |
| W521/A549V | ↑** | ↑*** | ↓** | ↑*** | ↑*** | ↓*** | ns | ↓** |
| W521/V649F | ↓*** | ↑** | ↓*** | ↑* | ↑*** | ↓* | ↑** | ↑* |
| G521Y | --^c^ | -- | -- | -- | -- | -- | ↑** | -- |
| Y521/G672A | ↓*** | -- | ↓*** | -- | ↓** | -- | ns | ↓** |

^a^ EQ (equilibrium of extracellular R6G), MAX (maximum slope [rate of R6G efflux]), and t_1/2_ (time to reach half maximum R6G efflux rate) are kinetic parameters of the R6G efflux activity of whole cells overexpressing the indicated Cdr1 variants (RFU = relative fluorescence units).

^b^ Arrows indicate increased or decreased values compared to wild-type (WT) Cdr1 or the parental Cdr1 mutants G521R, G521F, G521H, G521W, and G521Y, respectively. Statistically significant differences between them were evaluated with the Welch's t test (**p*<0.05, ***p*<0.01, ****p*<0.001, ns = not significant, -- = no data).

^c^ No detectable R6G transport of the Cdr1-G521Y variant.

**Supplementary Table S4.** DNA oligomer primers used in this study.

| **Primer** | **Sequence 5’-3’** | **Description** |
| --- | --- | --- |
| CDR1-G521D-for | CCGTTTTCGATCAACTTGTAATGGGTCTC | Cloning |
| CDR1-G521D-rev | GAGACCCATTACAAGTTGATCGAAAACGG | Cloning |
| CDR1-G521F-for | CCTATATTTTCCGTTTTCTTCCAACTTGTAATGGGTCTC | Cloning |
| CDR1-G521F-rev | GAGACCCATTACAAGTTGGAAGAAAACGGAAAATATAGG | Cloning |
| CDR1-G521W-for | CCTATATTTTCCGTTTTCTGGCAACTTGTAATGGGTCTC | Cloning |
| CDR1-G521W-rev | GAGACCCATTACAAGTTGCCAGAAAACGGAAAATATAGG | Cloning |
| CDR1-G521H-for | CCTATATTTTCCGTTTTCCATCAACTTGTAATGGGTC | Cloning |
| CDR1-G521H-rev | GAGACCCATTACAAGTTGATGGAAAACGGAAAATATAGG | Cloning |
| CDR1-G521Y-for | CCTATATTTTCCGTTTTCTATCAACTTGTAATGGGTCTC | Cloning |
| CDR1-G521Y-rev | GAGACCCATTACAAGTTGATAGAAAACGGAAAATATAGG | Cloning |
| AscI-for | GTTGGGCGCGCCCACACACATATATATAAGCC | Cloning |
| AscI-rev | GCCGGCCGCACTAGACTTGGCGCGCCTACCGTTCTTTTTAGGC | Cloning |
| Pdr5-upstream | GAGCATAAAACAGAGAGGCGATATAGG | Cloning |
| Pdr5-downstream | TATGAGAAGACGGTTCGCCATTCGGACAG | Cloning |
| CDR1-check | TGTCTGAAACCTTCTGTGGC | Sequencing reverse |
| CDR1-202 | ACCCATATGTCAGAAGTGCC | Sequencing forward |
| CDR1-846 | GGCAACCTATGGGTTATCAC | Sequencing forward |
| CDR1-1487 | AAGTGAGGTATGGTGTTGCG | Sequencing forward |
| CDR1-2112 | ATTCCACGGTCGTGAATTCC | Sequencing forward |
| CDR1-2676 | ATTGATGGGAGCATCTGGTG | Sequencing forward |
| CDR1-3301 | TATGGTGCTGATCCATGTCC | Sequencing forward |
| CDR1-3927 | TTCTGTCAATCCTCGAGGTG | Sequencing forward |


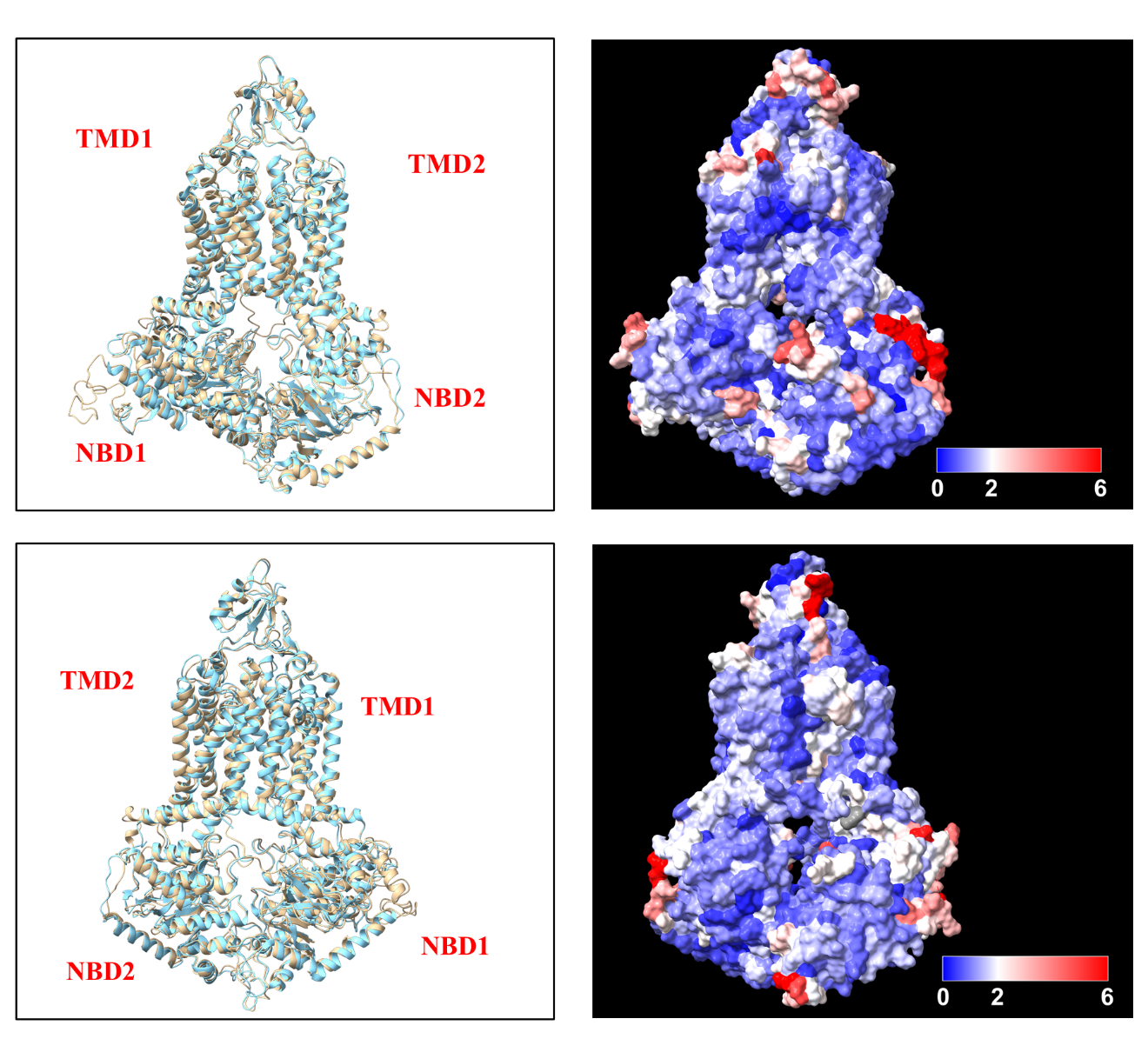
**Supplementary Figure S1.** Structural alignment of Cdr1 models. Left: Cartoon representation of the apo-state Cdr1 homology model based on Pdr5 (1) (yellow) and the cryo-EM structure of the Cdr1 apo protein (Protein Data Bank entry code 9IUK, (2)) (cyan). Right: Surface correlation maps with color gradients reflecting the Cα root-mean-square deviations (RMSD) between the two structures (0-6 Å; blue-white-red spectrum). Top and bottom panels are views from the front and the back of the transporter, respectively. TMD: transmembrane domain; NBD: nucleotide binding domain.


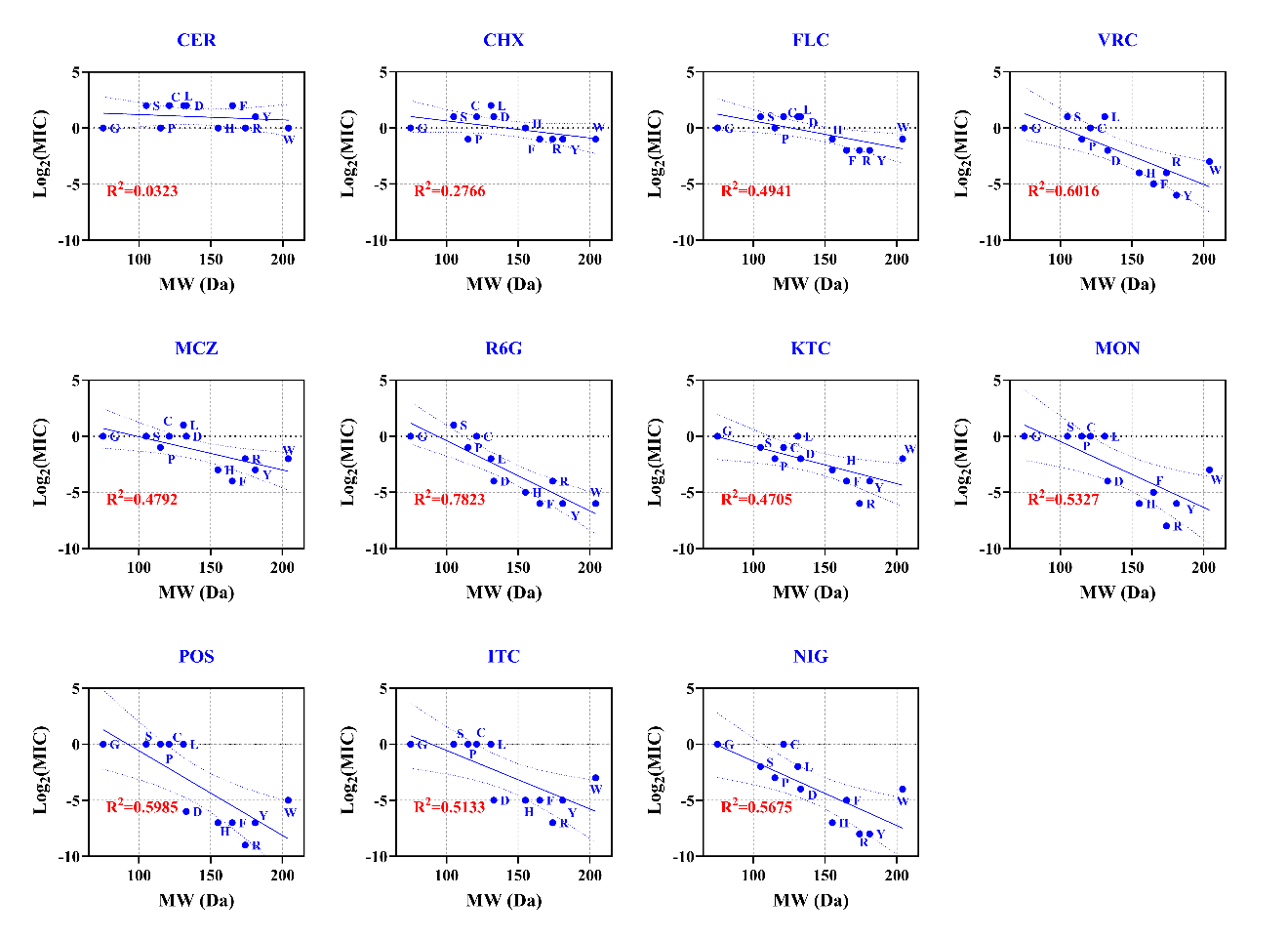
**Supplementary Figure S2.** Linear regression analysis of the log_2_ transformed values of the fold increased or decreased MIC values (relative to AD/CDR1B) for the 10 Cdr1-521 variants plotted against their molecular weights (MWs) (R² values for each of the 11 antifungal test substrates are shown in red adjacent to the trendlines). The 95% confidence intervals of the linear fits are represented by dashed blue boundaries. Individual data points are labeled with their respective mutant identifiers. CER: Cerulenin, CHX: Cycloheximide, FLC: Fluconazole, VRC: Voriconazole, MCZ: Miconazole, R6G: Rhodamine 6G, KTC: Ketoconazole, MON: Monensin, POS: Posaconazole, ITC: Itraconazole, NIG: Nigericin.


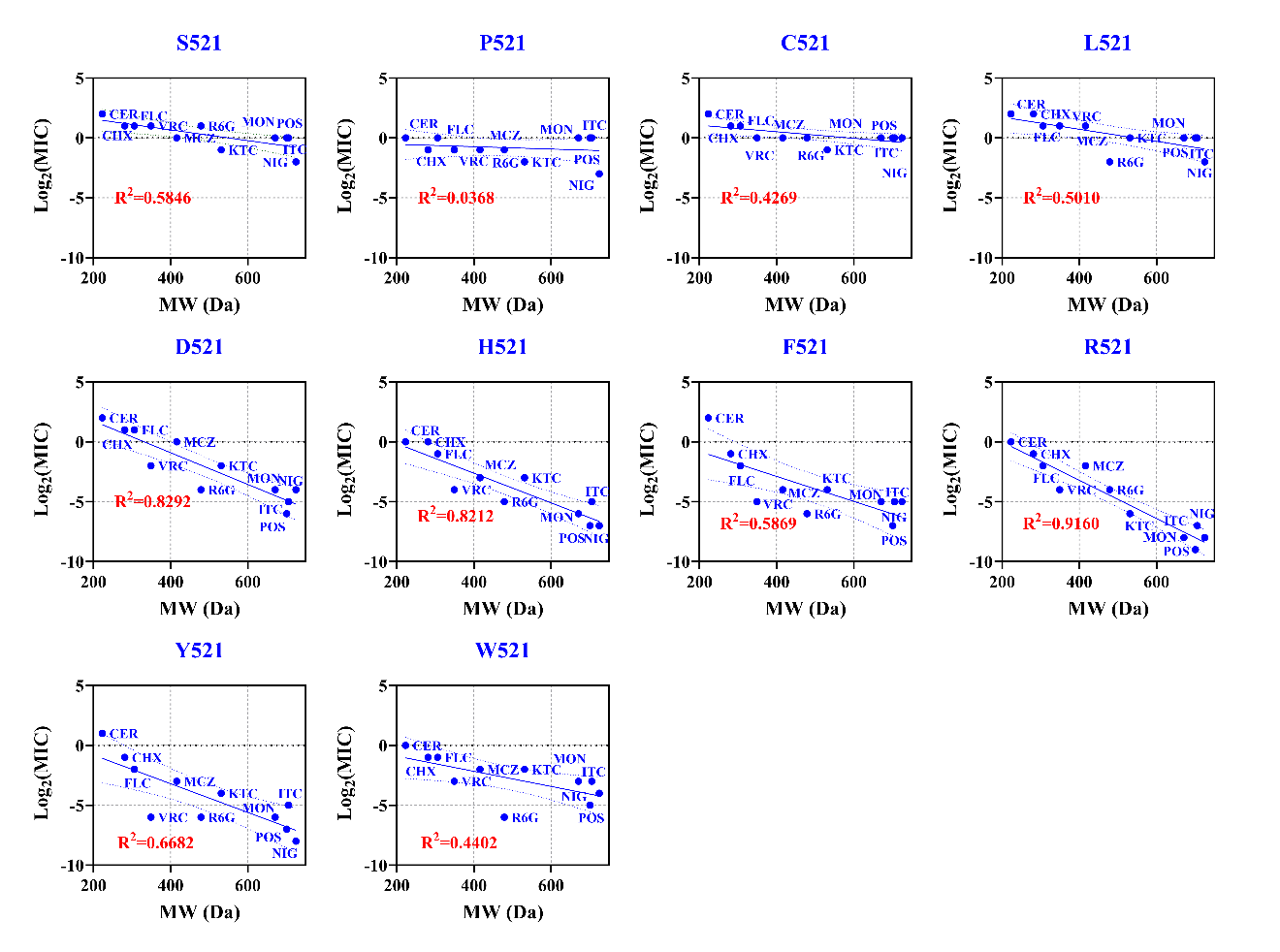
**Supplementary Figure S3.** Linear regression analysis of the log_2_ transformed values of the fold increased or decreased MIC values (relative to AD/CDR1B) for the 11 different antifungal test substrates plotted against their MWs (R² values for each of the 10 different Cdr1-G521 variants are shown in red adjacent to the trendlines). The 95% confidence intervals of the linear fits are represented by dashed blue boundaries. Individual data points are labeled with their respective substrate identifiers.


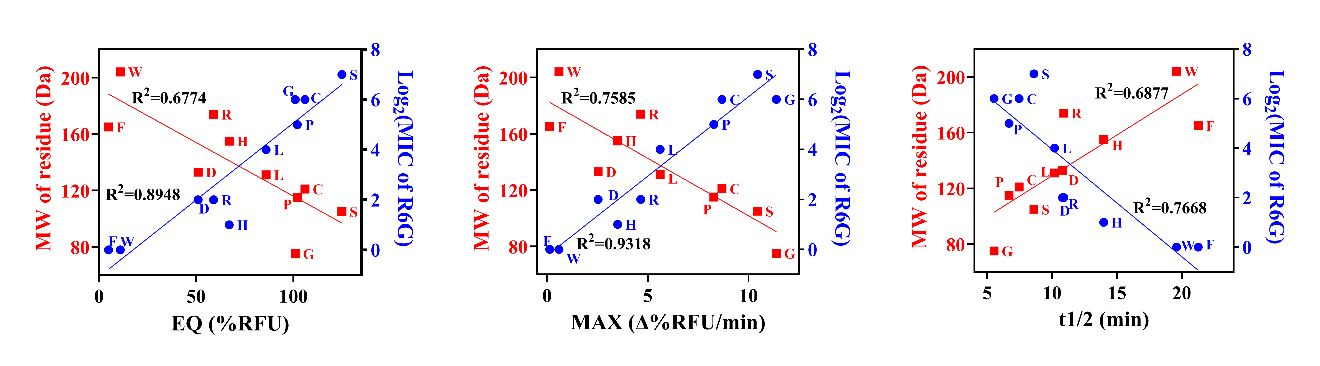
**Supplementary Figure S4.** Linear regression analysis for the three R6G efflux kinetic parameters and either the log_2_ transformed R6G MIC values (blue circles) or the MWs of the AD/CDR1B strain and Cdr1-G521 variants (red squares) (R² values are adjacent to the trendlines). Data points are labeled with corresponding mutant identifiers.


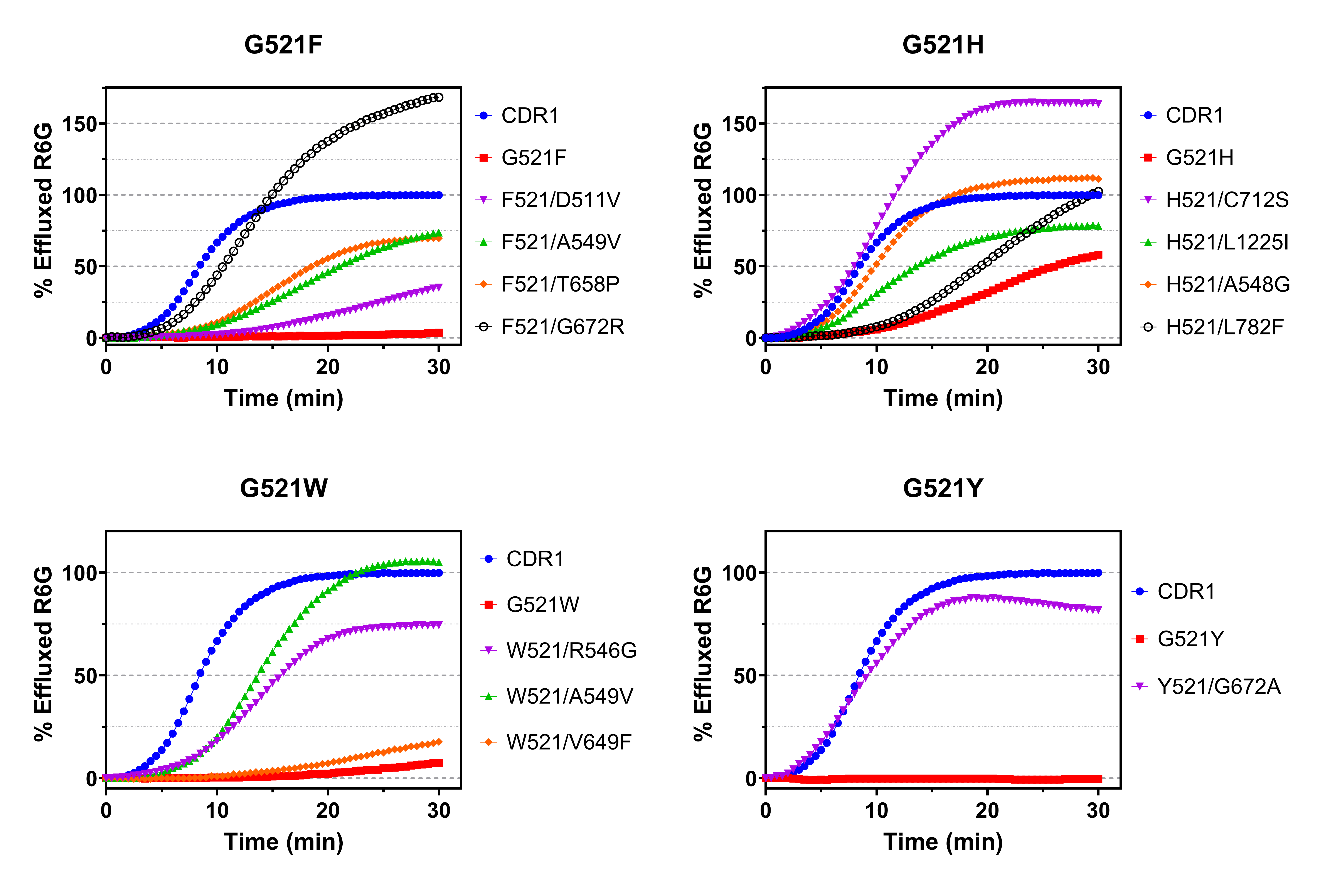


**Supplementary Figure S5.** Glucose-dependent R6G efflux by whole cells overexpressing Cdr1-G521F/H/WY variants or their suppressor mutants or the positive wild-type control Cdr1B. Extracellular R6G fluorescence was measured for 30 min at 30 s intervals post glucose addition (t=0). Data represent the means of two biological replicates. The R6G efflux values were normalized by i) subtracting the background signal of the negative control strain AD/pABC3, ii) adjusting for the different Cdr1 expression levels and iii) setting the extracellular R6G levels obtained by cells overexpressing Cdr1-G521 after 30 min incubation in the presence of glucose to 100%.

**References**

1. Harris A, Wagner M, Du D, Raschka S, Nentwig LM, Gohlke H, Smits SHJ, Luisi BF, Schmitt L. 2021. Structure and efflux mechanism of the yeast pleiotropic drug resistance transporter Pdr5. *Nat Commun* 12:5254.

2. Peng Y, Lu Y, Sun H, Ma J, Li X, Han X, Fang Z, Tan J, Qiu Y, Qu T, Yin M, Yan Z. 2024. Cryo-EM structures of *Candida albicans* Cdr1 reveal azole-substrate recognition and inhibitor blocking mechanisms. *Nat Commun* 15:7722.
